# Olig2 regulates p53-mediated apoptosis, migration and invasion of melanoma cells

**DOI:** 10.1038/s41598-021-87438-x

**Published:** 2021-04-08

**Authors:** Ji Eun Lee, Sungjin Ahn, Haengdueng Jeong, Seungchan An, Cheol Hwan Myung, Jeong Ah Lee, Sung Chan Hong, Youn Jin Kim, Jin Young Kim, Jong Hyuk Ryu, Minsoo Noh, Ki Taek Nam, Jae Sung Hwang

**Affiliations:** 1 Department of Genetic Engineering and Graduate School of Biotechnology, College of Life Sciences, Kyung Hee University, Yongin, 17104 Republic of Korea; 2 Natural Products Research Institute, College of Pharmacy, Seoul National University, 1 Gwanak-ro, Gwanak-gu, Seoul 08826 Republic of Korea; 3 Severance Biomedical Science Institute, Brain Korea 21 PLUS Project for Medical Science, Yonsei University College of Medicine, Seoul, Republic of Korea

**Keywords:** Cell death, Cell growth, Cell migration, Melanoma

## Abstract

Melanoma is a disease with a high recurrence rate and poor prognosis; therefore, the need for targeted therapeutics is steadily increasing. Oligodendrocyte transcription factor2 (Olig2) is a basic helix-loop-helix transcription factor that is expressed in the central nervous system during embryonic development. Olig2 is overexpressed in various malignant cell lines such as lung carcinoma, glioma and melanoma. Olig2 is known as a key transcription factor that promotes tumor growth in malignant glioma. However, the role of Olig2 in melanoma is not well characterized. We analyzed the role of Olig2 in apoptosis, migration, and invasion of melanoma cells. We confirmed that Olig2 was overexpressed in melanoma cells and tissues. Reduction of Olig2 increased apoptosis in melanoma cells by increasing p53 level and caspase-3/-7 enzyme activity. In addition, downregulation of Olig2 suppressed migration and invasion of melanoma cells by inhibiting EMT. Reduction of Olig2 inhibited expression of MMP-1 and the enzyme activity of MMP-2/-9 induced by TGF-β. Moreover, Olig2 was involved in the downstream stages of MEK/ERK and PI3K/AKT, which are major signaling pathways in metastatic progression of melanoma. In conclusion, this study demonstrated the crucial roles of Olig2 in apoptosis, migration, and invasion of melanoma and may help to further our understanding of the relationship between Olig2 and melanoma progression.

## Introduction

Malignant melanoma is a cancer originating from melanocytes, melanin-producing cells, and is one of the most aggressive types of cancer^1,2^. If melanoma is diagnosed early, the estimated five-year survival rate is as high as 98%. However, when melanoma spreads to the surrounding lymph nodes, the survival rate falls to around 60%; the survival rate is only about 20% when it spreads to distant organs. Although melanoma begins with a small nodule, its prognosis is poor because of its high metastasis rate after certain stages of growth^3,4^.

Apoptosis is a type of programmed cell death that can be seen in multi-cellular organisms due to internal biochemical changes^5^. When the level of DNA damage is so severe that repair is impossible, the p53 signaling system, which prevents cancer progression, is activated. B-cell lymphoma 2 (BCL-2) family proteins such as BCL-2 and BCL-2-associated X (BAX) are the main regulators of this intrinsic apoptosis pathway^6,7^. The apoptotic signal inhibits the expression of BCL-2 and thus forms the mitochondrial pore^8^. When cytochrome c is released into the cytoplasm through the mitochondrial pore, the apoptotic protease activation factor-1 (Apaf-1)/cytochrome c complex (called the apoptosome) is formed. This apoptosome activates caspase-3 or caspase-7, which then induce apoptosis^9,10^.

Epithelial-mesenchymal transition (EMT) is an important biological process by which epithelial cells lose their cell–cell junctions and polarity and obtain certain features of the mesenchymal phenotype, such as migratory and invasive behavior^11,12^. During EMT, transcription factors such as N-cadherin, Snail, Slug, and Zinc finger E-box-binding homeobox (ZEB) family are up-regulated and directly or indirectly repress transcription of E-cadherin. Subsequently, epithelial cells gain motility and invasiveness and then invade surrounding tissues^13,14^. As EMT is positively correlated with cancer metastasis, various cancer treatments have attempted to target EMT programs^15,16^.

Olig2 is a basic helix-loop-helix factor that is expressed in the central nervous system during embryonic development^17,18^. Olig2 is expressed in multipotent neurospheres from the murine embryonic forebrain, where it promotes proliferation of neural progenitors^19^. Although Olig2 was thought to be expressed only in neural tissue, Ying-Wei Lin et al. found that it is expressed in various tissues including kidney, thymus, lung, brain, and skin. Additionally, they found overexpression of Olig2 in malignant cell lines from lung carcinoma, breast cancer, and melanoma^20^. However, the role of Olig2 in melanoma is not well characterized. Our data provide the first evidence supporting the role of Olig2 in invasive growth of melanoma. Here, we investigated whether Olig2 regulates melanoma progression. We show that Olig2 is highly expressed in various melanoma cell lines and contributes to invasive growth of melanoma cells.

## Results

### Olig2 expression is elevated in human melanoma

**Figure 1:**
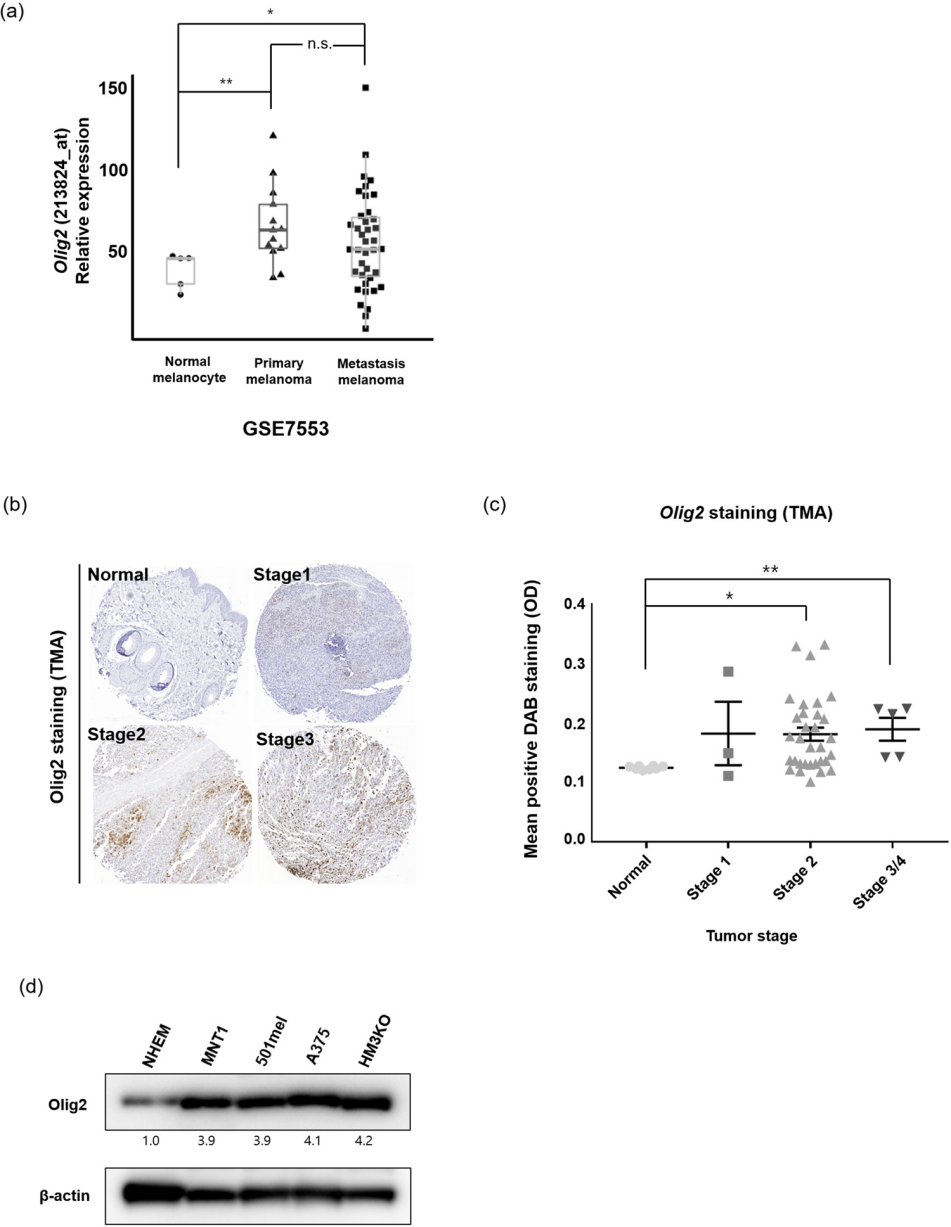
Olig2 was upregulated in melanoma. (**a**) The levels of Olig2 was determined by analysis of GSE7553 public dataset containing the gene expression of normal melanocyte (n = 4), primary melanoma (n = 14) and metastatic melanoma (n = 40). The data was analyzed using Wilcoxon signed-rank tests (ns: not significant, *p < 0.05; **p < 0.01 versus Normal) (**b**) IHC analysis of Olig2 in normal skin (n = 8) and malignant melanoma skin (n = 40) tissue sample was performed and (**c**) the representative graph was shown. *p* values were analyzed using unpaired Student’s t-test (*p < 0.05; **p < 0.01) (**d**) Normal human melanocyte (NHEM) and four human metastatic melanoma cells were lysed with lysis buffer. Olig2 protein expression was detected by western blot analysis with Olig2 (H-10) antibody.

To investigate the clinical significance of Olig2 in melanoma tissue, we analyzed published microarray data of Olig2 from the GSE7553 data set. We found that expression of Olig2 was elevated in primary and metastatic melanoma tissues compared with normal melanocyte tissues. However, there was no significant difference in the expression level of Olig2 between primary and metastatic melanoma (Fig. 1a). We performed Olig2 IHC analysis on a melanoma tissue microarray (TMA). Olig2 staining was higher in stage 2 and 3/4 melanoma tissue compared with normal skin tissue (Fig. 1b,c). To examine the basal expression of Olig2, we performed immunoblotting in normal human epidermal melanocytes (NHEM) and four human metastatic melanoma cell lines (MNT-1, 501mel, A375, and HM3KO). Olig2 was expressed significantly higher in the four metastatic melanoma cell lines compared with NHEM. There was no significant difference in the expression of Olig2 between the four melanoma cell lines (Fig. 1d).

### Reduction of Olig2 inhibits growth of melanoma cells

**Figure 2:**
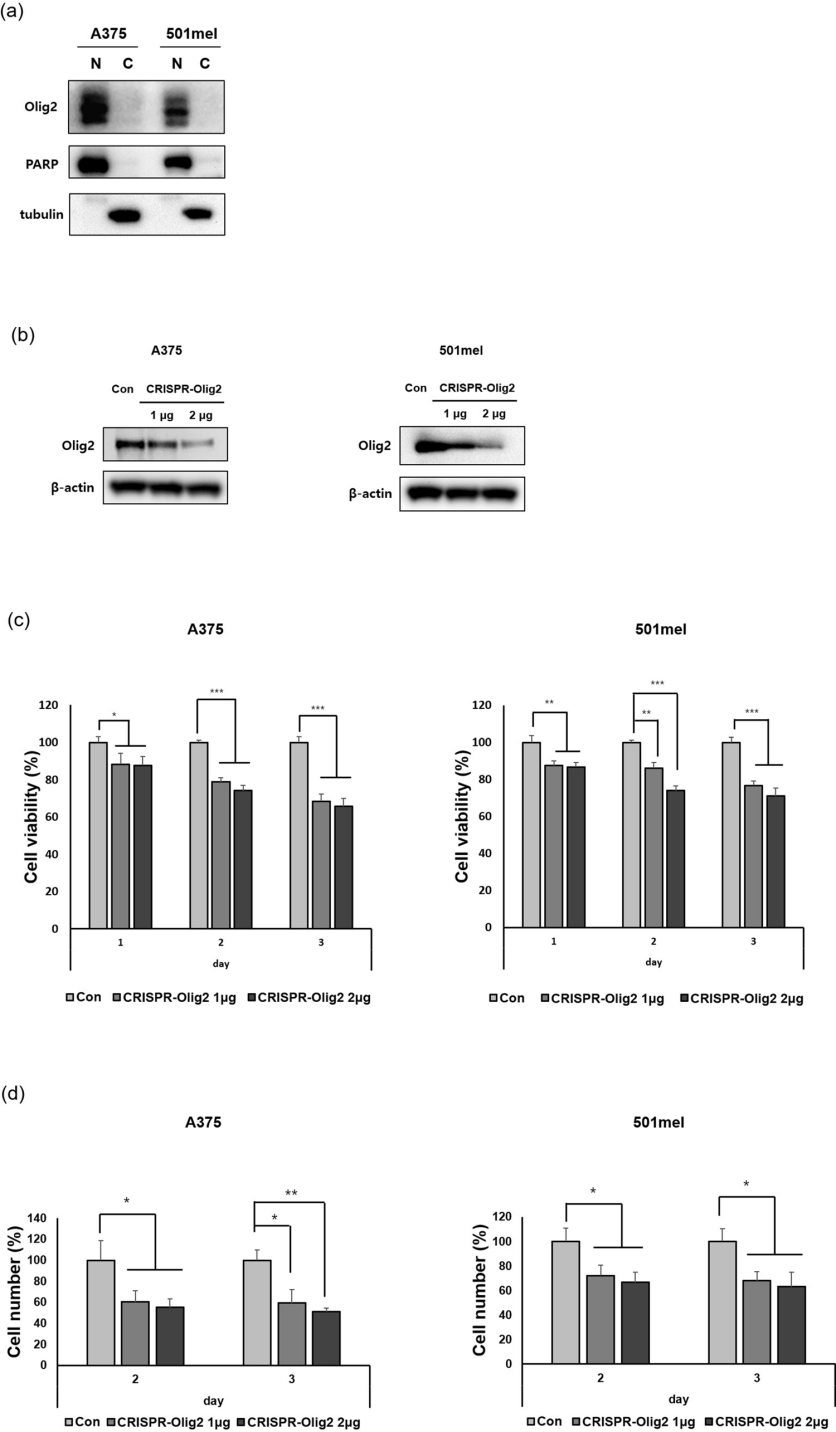
Downregulation of Olig2 decreased viability of melanoma cells. (**a**) Nuclear and cytoplasmic extracts were analyzed by western blot analysis using Olig2 (AF2418) antibody. (**b**) Cells were transfected with negative control (Con) or CRISPR/Cas9-Olig2 plasmid (CRISPR-Olig2) (1 μg and 2 μg, respectively). Western blot analysis of Olig2 protein expression was performed with Olig2 (H-10) antibody. Original images of blots were provided in supplementary file. (**c**) Cells were transfected with negative control (Con) or CRISPR/Cas9-Olig2 plasmid (CRISPR-Olig2) (1 μg and 2 μg, respectively). Cell viability was determined by EZ-Cytox assay 24 h, 48 h and 72 h after transfection respectively. Results are presented as percentages of the Con and the data were analyzed using Student's unpaired t-tests (*p < 0.05; **p < 0.01; ***p < 0.001 versus Con cells, n = 3). (**d**) 48 and 72 h after transfection, cell number was measured by trypan blue exclusion. Results are presented as percentages of the Con and the data were assessed using Student's unpaired t-tests (*p < 0.05; **p < 0.01; ***p < 0.001 versus Con cells, n = 3).

We examined Olig2 localization using western blot analysis of nuclear and cytoplasmic fractions from melanoma cells. Olig2 predominantly localized in the nucleus in both A375 and 501mel melanoma cells (Fig. 2a).

To identify the roles of Olig2 in melanoma, we used a CRISPR/Cas9 system to precisely create a loss-of-function mutation in the Olig2 gene. Our results showed successful dose-dependent inhibition of CRISPR-Olig2 in A375 and 501mel melanoma cells (Fig. 2b). Next, we examined cell viability after transfection of CRISPR-Olig2 to examine the role of Olig2 in melanoma survival. Down-regulation of Olig2 decreased cell viability in A375 and 501mel cells (Fig. 2c). We further validated these results by cell counting assays. As shown in Fig. 2, knockdown of Olig2 decreased the number of melanoma cells (Fig. 2d).

### Olig2 inhibition induces apoptosis of melanoma cells

**Figure 3:**
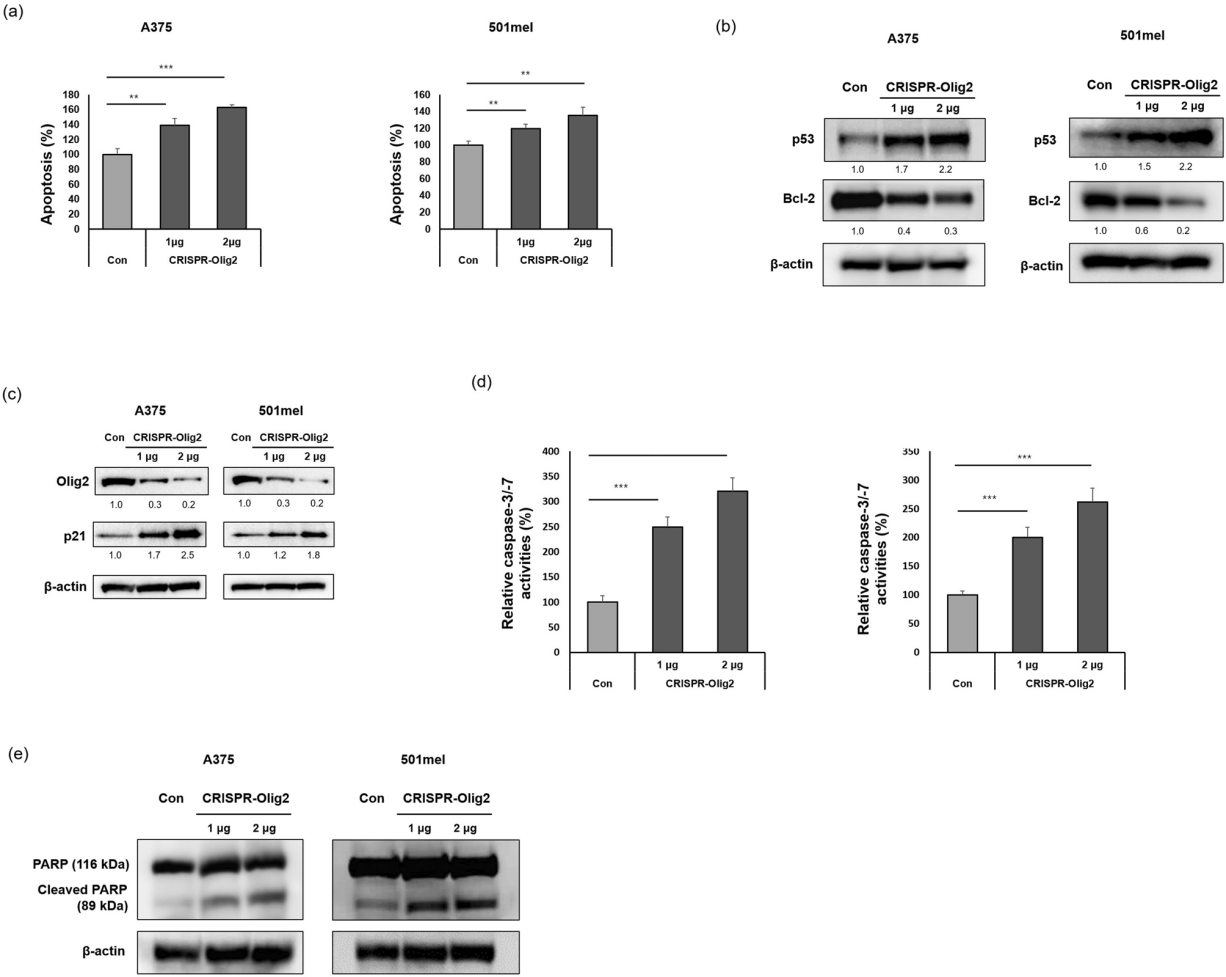
Downregulation of Olig2 induced p53-mediated apoptosis. (**a**) Apoptotic cells were detected using RealTime Glo Annexin V apoptosis assay kit. 24 h after transfection, the intensity of luminescence was recorded using a luminescence plate reader (**p < 0.01; ***p < 0.001 versus Con cells, n = 3). (**b**) Protein expression of Olig2, p53 and Bcl-2 was detected in A375 and 501mel melanoma cells by western blot analysis with Olig2 (H-10) antibody. β-actin was used as loading control. The band intensity was quantified using NIH ImageJ software 1.45 s. (**c**) Protein expression of Olig2 and p21 in A375 and 501mel melanoma cells by western blot analysis. (**d**) The activities of caspases-3/-7 were evaluated using Caspase-Glo 3/7 assay kit. 24 h after transfection, fluorescence intensity was detected at 485 nm excitation and 520 nm emission on a fluorescence plate reader (***p < 0.001 versus Con cells, n = 3). (**e**) PARP and cleaved PARP expression in two melanoma cells transfected with negative control (Con) or CRISPR/Cas9-Olig2 plasmid (CRISPR-Olig2) (1 μg and 2 μg, respectively) was detected by western blot analysis. Original images of blots were provided in supplementary file.

To examine the mechanism by which silencing of Olig2 suppressed melanoma cell viability, we measured cell apoptosis following Olig2 knockdown. As shown in Fig. 3a, reduction of Olig2 induced apoptosis of both A375 and 501mel melanoma cells. Next, we detected apoptosis-related factors such as p53, p21 and Bcl-2 after transfection of CRISPR-Olig2 in two melanoma cell lines. We found that reduction of Olig2 increased p53 and p21 expression, and decreased Bcl-2 expression (Fig. 3b,c). To determine whether downregulation of Olig2 activates caspases involved in apoptosis, we assessed caspase-3/-7 enzymatic activity using a fluorogenic substrate that can detect this enzymatic reaction in cells. When Olig2 level was decreased, caspase-3/-7 activity increased in melanoma cells (Fig. 3d). Subsequently, we observed cleavage of PARP, which is considered an apoptotic marker. Western blot analysis revealed that downregulation of Olig2 induced cleavage of PARP (89 KD) in A375 and 501mel cells (Fig. 3e). Taken together, these findings indicate that reduction in Olig2 induces apoptosis in melanoma cells.

### Silencing of Olig2 inhibits migration and invasion of melanoma cells by regulating EMT-related factors

**Figure 4:**
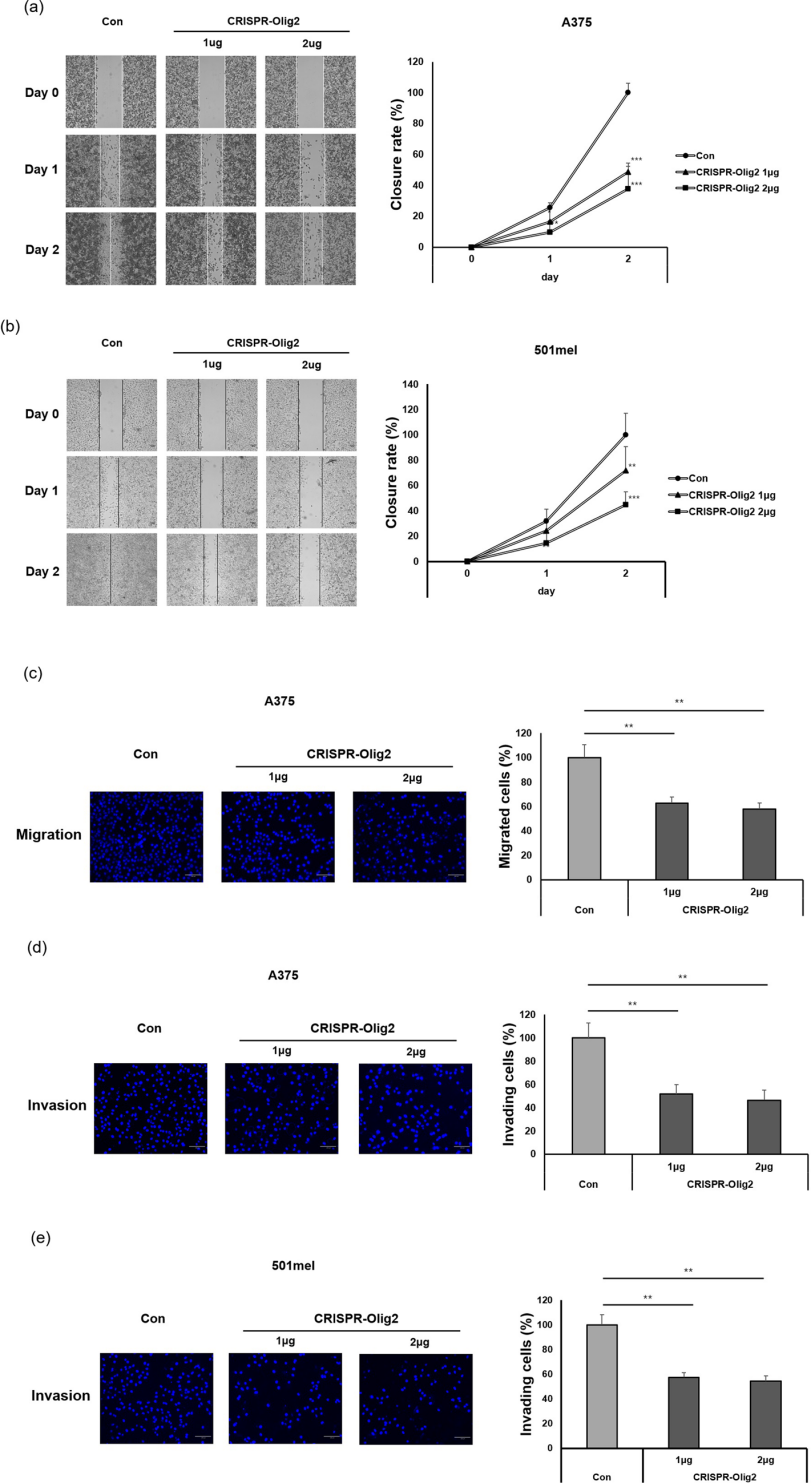
Olig2 knockdown decreased migration and invasion of melanoma cells. Wound healing assay was performed to evaluate the migration abilities of (**a**) A375 cells and (**b**) 501mel cells after silencing of Olig2 expression. The cell migration into the wound was measured at 24 h and 48 h after scratch. Representative images (magnification, × 40) of cells were taken celenas digital imaging system. Scale bar: 100 µm. The migration was determined by the rate of cells filling the scratched area (*p < 0.05; **p < 0.01; ***p < 0.001 versus Con cells, n = 3). Transwell assay was performed to evaluate the migration ability of (**c**) A375, and invasion ability of (**d**) A375 and (**e**) 501mel cells after silencing of Olig2 expression. The cells which migrated or invaded through the membrane were stained with DAPI for visualization of the nuclei. Representative images and quantitative analysis of migrating or invading cells (*p < 0.05; **p < 0.01 versus Con cells, n = 3).

Metastasis, which is the main characteristic of malignant melanoma, is a phenomenon in which cells are separated from a primary tumor and relocate^21^. To investigate whether Olig2 regulates migration of melanoma cells, we evaluated cell migration using an in vitro scratch assay. As shown in Fig. 4a,b, cells closed the wound more slowly in the CRISPR-Olig2 transfection groups than in the control groups. To further examine the metastatic role of Olig2, we examined the migration and invasion of cells by performing a transwell assay using a chamber with 8-µm pores. The results revealed that the number of cells that migrated or invaded the underside of the chamber decreased in the CRISPR-Olig2 transfection groups compared to the control groups (Fig. 4c–e).

**Figure 5:**
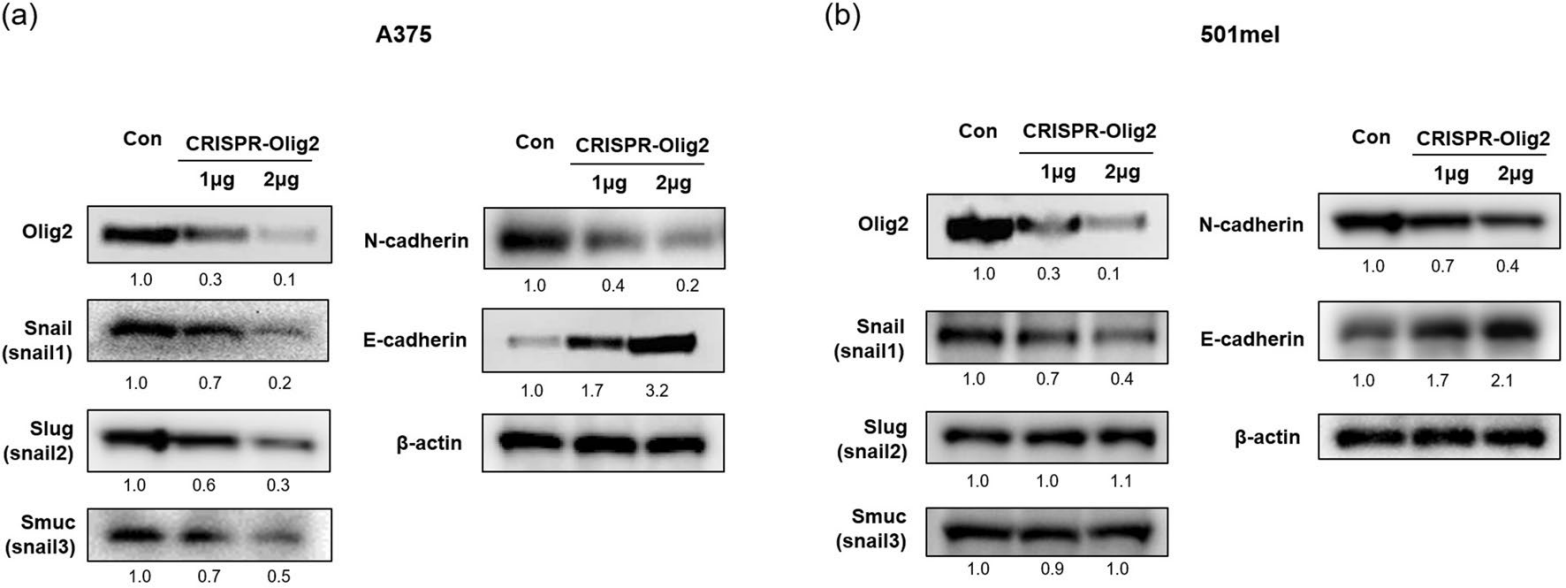
Olig2 regulated the EMT markers in melanoma cells. The expression of EMT markers including Snail, Slug, Smuc, N-cadherin and E-cadherin was detected in (**a**) A375 cells and (**b**) 501mel cells using by western blot analysis with Olig2 (H-10) antibody. The band intensity was quantified using NIH ImageJ software 1.45 s and normalized relative to β-actin. Original images of blots were provided in supplementary file.

EMT is a biological mechanism of cancer cell recurrence and metastasis, accompanied by a loss of epithelial proteins such as E-cadherin and occludin, and an increase in mesenchymal proteins including N-cadherin, vimentin, and smooth muscle actin^22^. To further define the role of Olig2 as an EMT regulator, we examined the expression level of EMT-related factors by western blot analysis. Knockdown of Olig2 led to simultaneous downregulation of Snail (Snail1), Slug (Snail2), Smuc (Snail3) and N-cadherin, and upregulation of E-cadherin in A375 cells. In addition, these changes in protein expression were dependent on the concentration of CRISPR-Olig2 treatment (Fig. 5a). In 501mel cells, unlike A375 cells, Slug and Smuc expression did not change when Olig2 was decreased. However, Snail and N-cadherin expression was reduced and E-cadherin was increased in a concentration-dependent manner (Fig. 5b).

### Olig2 regulates MMP-2/-9 activation and MMP-1 expression

**Figure 6:**
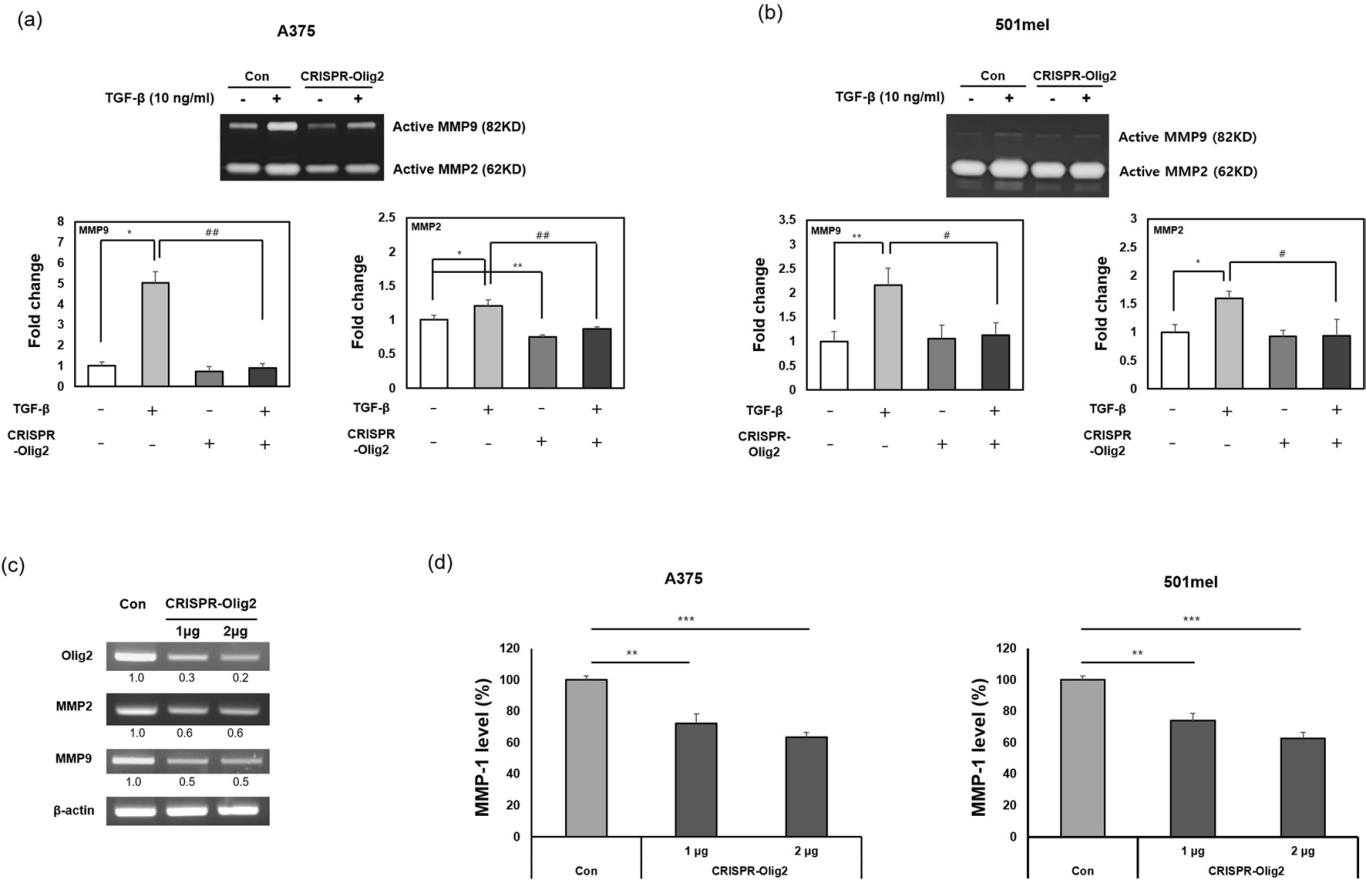
Downregulation of Olig2 decreased MMP-2/-9 activities and MMP-1 expression. Gelatin zymography assay was performed with the conditioned media from (**a**) A375 and (**b**) 501mel cells transfected with negative control (Con) or CRSPR/Cas9-Olig2 plasmid (CRISPR-Olig2) followed by TGF-β (10 ng/ml) treatment. The fold-change in MMP-2 and MMP-9 activities was assessed by densitometry compared to Con group. The band intensity of MMP-2 and MMP-9 was quantified using NIH ImageJ software. (*p < 0.05; **p < 0.01versus Con group and #*p* < 0.05 versus TGF-β group, n = 3). (**c**) MMP-2 and MMP-9 mRNA were evaluated using each primer by RT-PCR. The band intensity was quantified using NIH ImageJ software and normalized relative to β-actin. Original images of gels were provided in supplementary file. (**d**) The concentration of MMP-1 was measured by ELISA using the supernatant of two melanoma cells. (**p < 0.01; ***p < 0.001 versus Con group, n = 3).

Since matrix metalloproteinase-2/-9 (MMP-2/-9) are known to play an important role in melanoma metastasis, we estimated their enzymatic activity with gelatin zymography. MMP-2/-9 activity was increased following treatment with transforming growth factor-β (TGF-β). However, the levels of MMP-2/-9 were reduced in the Olig2-deficient group compared to the TGF-β treatment group in both A357 and 501mel cells (Fig. 6a,b). In addition, reduction of Olig2 inhibited mRNA levels of MMP-2 and MMP-9 (Fig. 6c). We further verified that Olig2 influences the expression of MMP-1, another MMP that is important for invasion and growth of melanoma. An ELISA assay showed that expression of MMP-1 was decreased in the CRISPR-Olig2 transfection group (Fig. 6d).

### Active MEK/ERK and PI3K/AKT signaling controls Olig2 protein expression

**Figure 7:**
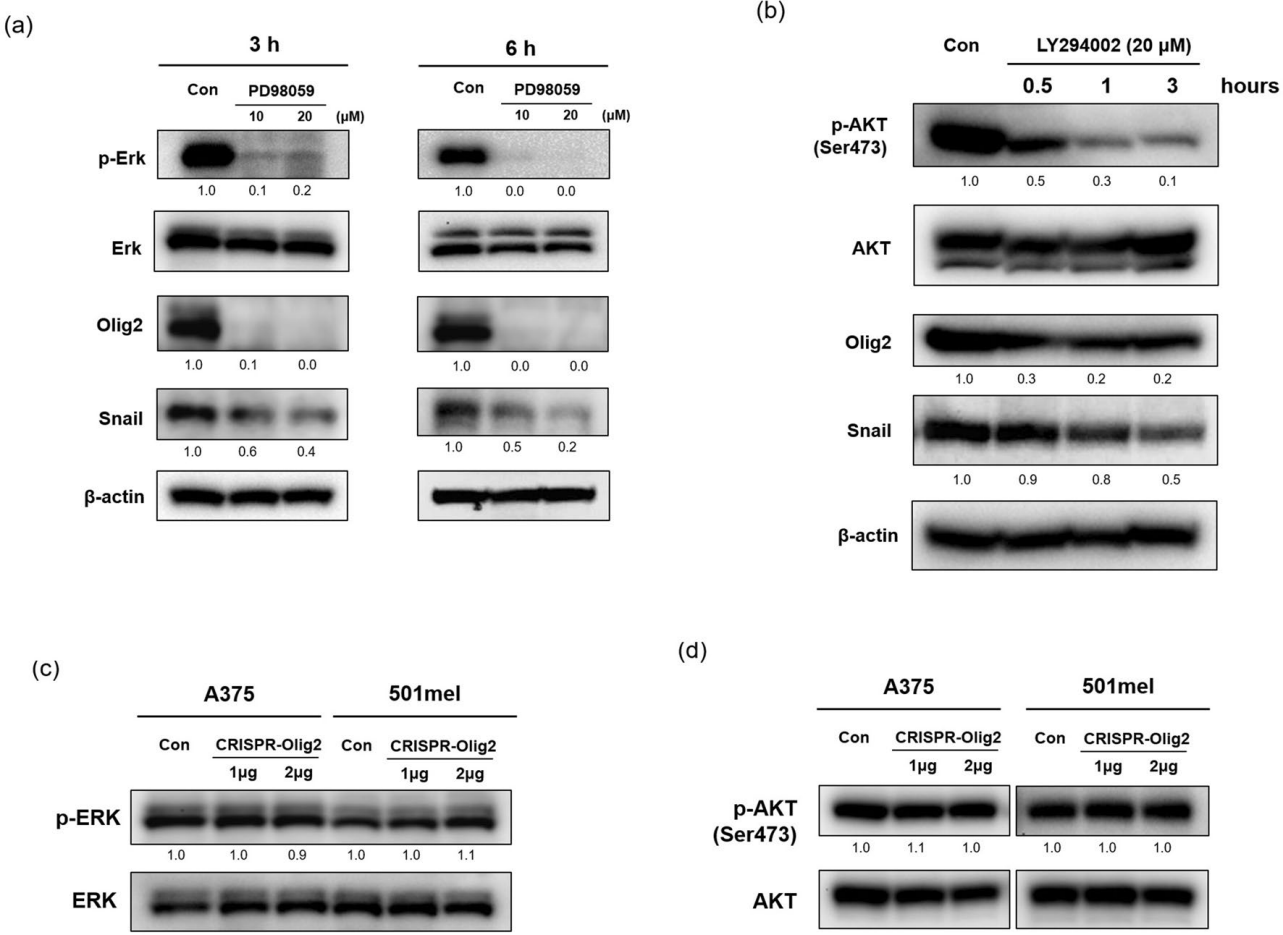
The MEK inhibitor (PD98059) and PI3K inhibitor (LY294002) decreased expression of Olig2. (**a**) A375 cells were treated with PD98059 for 3 h and 6 h. Protein expression of p-ERK, ERK, Olig2 (H-10) and Snail was detected by western blot analysis. (**b**) 501mel cells were treated with LY294002 for the indicated times. Protein expression of p-AKT, AKT, Olig2 (H-10) and Snail was detected by western blot analysis. (**c**) A374 and (**d**) 501mel cells were transfected with negative control (Con) and CRISPR/Cas9-Olig2 plasmid (CRISPR-Olig2). After 24 h transfection, western blot analysis was performed to identify the expression of p-ERK1/2, ERK, p-AKT and AKT. Original images of blots were provided in supplementary file.

Hyperactivation of the RAS-RAF-MAPK pathway is closely associated with development of melanoma. Extracellular signal-regulated kinase (ERK) activity in cutaneous melanoma is mostly caused by mutations in BRAF, the most common genetic variation in melanoma^23^. To determine whether Olig2 is a downstream effector of mitogen-activated protein kinase kinase (MEK)/ERK signaling, we treated A375 cells with PD98059, an MEK inhibitor. PD98059 markedly suppressed activation of ERK and expression of Olig2 and Snail (Fig. 7a). In melanoma, the Phosphoinositide 3-kinase (PI3K)/AKT pathway is implicated in mutations in phosphatase and tensin homologue (PTEN) and NRAS, which occur most frequently after BRAF mutations. Because NRAS is located upstream of BRAF, NRAS mutations activate mitogen-activated protein kinase (MAPK) and the PI3K/AKT/ mammalian target of rapamycin (mTOR) pathway^24^. Therefore, we next identified whether Olig2 is a downstream effector of PI3K/AKT. We found that phosphorylation of AKT and expression of Olig2 and Snail decreased after treatment with the PI3K inhibitor LY294002 (Fig. 7b). Additionally, we identified the roles of Olig2 in ERK and AKT activation. However, when cells were transfected with CISRPR-Olig2, there was no change in the activity of ERK or AKT (Fig. 7c,d).

### Olig2 silencing further increased the inhibition of cell viability by dabrafenib in 501mel cells

**Figure 8:**
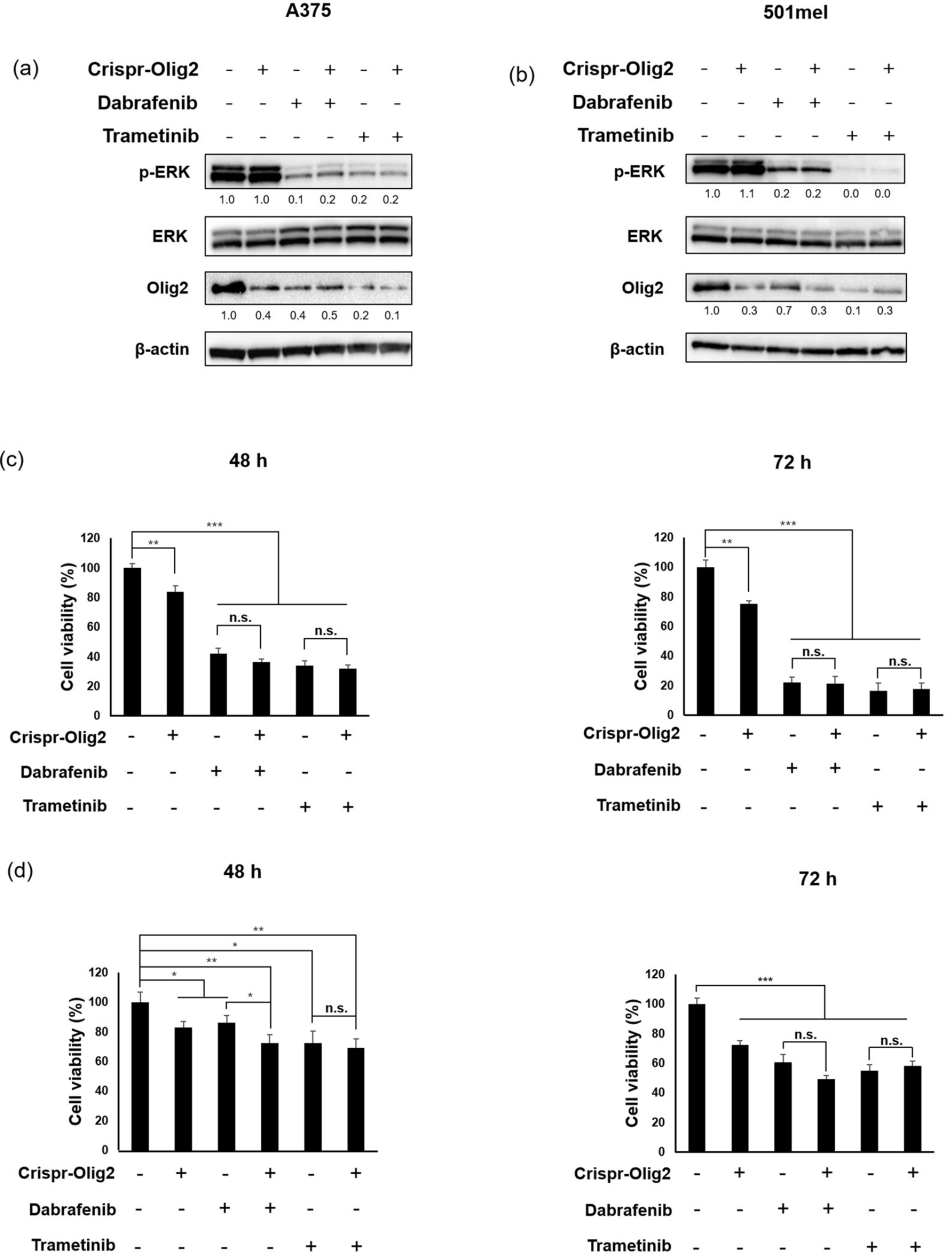
Combinational effects of Olig2 silencing and BRAF inhibitor (dabrafenib) or MEK inhibitor (trametinib) on melanoma cells. (**a**, **b**) A375 and 501mel cells were incubated with CRISPR-Olig2, dabrafenib (50 nM), trametinib (5 nM), or their combinations for 24 h. Ratio of phospho-ERK/total ERK and Olig2 (H-10)/β-actin were indicated below each lane and obtained by densitometry using the ImageJ software. Original images of blots were provided in supplementary file. Viability of A375 (**c**) and 501mel (**d**) was determined by EZ-Cytox assay 48 h and 72 h after treatment respectively. Results are presented as percentages of the Con and the data were analyzed using Student's unpaired t-tests (*p < 0.05; **p < 0.01; ***p < 0.001, n = 3).

We next investigated whether combined treatment of BRAF inhibitor (dabrafenib) or MEK inhibitor (trametinib) with CRISPR-Olig2 could be effective in reducing melanoma cell viability. Dabrafenib and trametinib inhibited phosphorylation of ERK or AKT, and decreased expression of Olig2 (Fig. 8a,b) Co-treatment of CRISPR-Olig2 and dabrafenib slightly reduced cell viability compared to the treatment of dabrafenib alone in 501mel cells. However, combined treatment with CRISPR-Olig2 and trametinib showed no significant difference from trametinib alone in 501mel cells. Also, no synergistic effect was observed in A375 cells (Fig. 8c,d).

## Discussion

Malignant melanoma develops more frequently than any other tumor and can spread to most parts of the body. Therefore, prevention and treatment of melanoma are increasing in importance; accordingly, studies on the cell biology of tumor development and immune evasion of melanoma are ongoing^25^. Olig2 has been studied in certain types of neuronal and glial cells for its roles in tumorigenicity. In glioma stem cells, Olig2 promotes cell proliferation through direct inhibition of *p21*^*WAF1/CIP1*^, a tumor suppressor. Phosphorylation of the serine motif of Olig2 promotes proliferation of neural progenitor cells and induces potential astrocytoma malignancies^19,26^. Recently, Olig2 was shown to increase in recurrent medulloblastoma after radiation therapy and in drug-resistant regions^27^. However, the role of Olig2 in melanoma is still not clearly understood.

Here, we identified that Olig2 mRNA expression was high in primary and metastatic melanoma tissues compared with normal human melanocyte tissues by microarray analysis. In addition, we verified that metastatic melanoma has higher Olig2 protein level than normal human melanocytes and normal skin (Fig. 1a–d). To investigate the function of Olig2 in melanoma progression, we used CRISPR/Cas9 to knockdown the Olig2 gene in A375 and 501mel metastatic melanoma cells. Previous studies found that Olig2 controls cell cycling and growth in malignant neural progenitors and plays an important role in glioma formation^19^. We found that knockdown of Olig2 suppressed survival of A375 and 501mel cells and decreased the number of cells (Fig. 2c,d). These findings suggest that Olig2 might be positively correlated with melanoma progression.

Previously, it was reported that Olig2 suppressed p53 response in neural progenitors and gliomas^28^. Consistent with this result, our study showed that knockdown of Olig2 increased expression of p53 and induced apoptosis of two melanoma cell lines (Fig. 3a,b). Suppression of Olig2 increased the protein expression of p21, confirming the possibility that Olig2 may be associated with p21 in melanoma (Fig. 3c), as in Ligon, K. L. et al*.*’s study that Olig2 directly inhibits p21^19^. The Bcl-2 gene family, which is normally involved in anti-apoptotic activities, can potentially cause cancer, and cancer-related proteins such as Myb and Ras induce high levels of Bcl-2 in hematopoietic cells. Therefore, Bcl-2 is considered an inhibitor of apoptotic proteins and may be an important target for cancer treatment^29^. We found that reduction of Olig2 in melanoma cells inhibited the expression of Bcl-2 (Fig. 3b). In addition, Olig2 knockdown induced activation of caspase-3/-7 (Fig. 3d) and PARP cleavage (89 KD) (Fig. 3e). Taken together, these findings suggest that Olig2 downregulation promoted a p53-mediated apoptosis pathway, suggesting that low expression of Olig2 is associated with unfavorable survival in melanoma cells. Microphthalmia-associated transcription factor (MITF) is a key transcription factor that regulates survival and invasiveness of melanoma^30^. To determine whether Olig2 regulates MITF expression, we inhibited Olig2 in two melanoma cells and then identified the protein expression of MITF by immunoblotting. However, Olig2 downregulation didn’t induce significant change in expression of MITF. Thus, Olig2 is expected to regulate survival and migration independently of MITF (Fig. S1).

Along with overgrowth, the most dangerous aspect of cancer is metastasis. Melanoma penetrates deep into the skin layer and spreads to lymph nodes, blood vessels, and even distal parts of the body such as bone, brain, liver, and lung^31^. The association between Olig2 and metastasis is unknown. Therefore, we examined the wound healing assay after suppressing Olig2 expression to determine whether Olig2 is involved in melanoma migration. We confirmed that movement of cells into the wound slowed when Olig2 expression was inhibited in A375 and 501mel cells (Fig. 4a,b). Additionally, in the transwell migration and invasion assay, reduction of Olig2 inhibited the migration and invasion of A375 and 501mel cells (Fig. 4c–e). These results indicate that Olig2 is required for migration and invasion in melanoma cells.

In addition to embryonic development, EMT provides a model for studies in which carcinoma is dedifferentiated and more malignant. If the EMT program is pathologically activated in a cancerous environment, it can lead to cancer metastasis^12^. As the transcription factor Snail can inhibit E-cadherin expression, it has been recognized as a key factor in EMT. E-cadherin and N-cadherin are important regulators of morphogenesis in mouse embryonic formation and are considered representative cadherins in epithelial and mesenchymal tissues^22^. We further investigated the mechanistic roles of Olig2 in melanoma invasion through the expression of EMT markers. Western blot analysis revealed that decreased Olig2 inhibited the expression of EMT-inducing markers including Snail, Slug, Smuc, and N-cadherin and conversely increased the expression of E-cadherin in A375 cells. Depending on the cell type, the regulation of Slug and Smuc by Olig2 may appear differently. In 501mel cells, only the expression of Snail, N-cadherin, and E-cadherin changed when Olig2 decreased (Fig. 5). Because melanoma cells are derived from embryonic neural crest, not epithelial cell origin, they do not undergo traditional EMT. Instead, melanoma cells appear EMT-like process by which they acquire mesenchymal or epithelial features. Our results suggest that Olig2 control EMT-like process in melanoma cells.

MMPs are endogenous factors that regulate proteolysis and play an important role in degradation of extracellular matrix. They are normally involved in important functions such as wound healing or embryonic development; however, they are also involved in pathological processes such as cancer metastasis and tumor growth. In particular, MMP-2/-9 are key regulators of cancer invasion^32,33^. They break down type IV collagen and fibronectin, which are important constituents of the basement membrane, and contribute to angiogenesis, which provides nutrients for tumor growth^34^. Additionally, MMP-1, known as pro-collagenase-1, is overexpressed in BRAF-mutated melanoma and contributes to melanoma invasion and growth by activating the ERK pathway^35^. Blackburn et al. also found that the MMP-1/protease activated receptor-1 (PAR1) axis promotes progression of melanoma by inducing the transition from the non-invasive radial-growth-phase (RGP) phenotype to the invasive vertical growth phase (VGP) phenotype^36^. MMP-1 expression is associated with poor prognosis in colorectal cancer. Specific inhibitors of MMP-1 suppressed tumor induction in mouse model of colon cancer^37,38^. Therefore, inhibiting the expression of MMP-2, MMP-9, and MMP-1 is important to prevent tumor invasion and metastasis. In our study, Olig2 knockdown suppressed mRNA expression of MMP-2/-9 and TGF-β-induced MMP-2/-9 activity (Fig. 6a–c). Furthermore, the expression level of MMP-1 was attenuated in Olig2 knockdown of melanoma cells (Fig. 6d). These results suggest that Olig2 can regulate melanoma invasion through activation of MMPs and might be associated with TGF-β-induced EMT in melanoma cells.

BRAF and NRAS mutations are representative genetic mutations that cause skin melanoma. They primarily activate MAPK and the PI3K/AKT/mTOR signaling pathway^39,40^ and cause various transcription factors such as cAMP-response element binding protein (CREB) and Brn-2 (POU3F2) to become increasingly active, resulting in excessive cell proliferation^41,42^. Therefore, the BRAF inhibitors vemurafenib and dabrafenib have been approved by the FDA for treatment of melanoma. However, the leading cause of death in patients with advanced melanoma is persistent recurrence and chemotherapy resistance. We demonstrated that Olig2 is associated with MEK/ERK and PI3K/AKT signaling pathways in melanoma cells. Treatment with PD98059 significantly suppressed expression of Olig2 and Snail in melanoma cells (Fig. 7a). Also, LY294002 decreased the levels of Olig2 and Snail (Fig. 7b). However, CRISPR-Olig2 had no effect on ERK or AKT activation (Fig. 7c,d). These results suggest that Olig2 is a downstream target of MAPK and PI3K pathway and may be involved in EMT regulation through activation of MAPK and PI3K signaling pathways. Cell viability slightly decreased from combining CRISPR-Olig2 and dabrafenib compared to dabrafenib alone in 501mel cells. However, it appears that block of Olig2 and BRAF&MEK inhibition do not show a distinct synergistic effect in reducing the viability of melanoma cells (Fig. 8c,d). This is probably the result of suppressing Olig2 expression by inhibitors treatment as in Fig. 8a,b. Further in vivo studies are necessary to confirm the roles of Olig2 in metastatic progression of melanoma.

**Figure 9:**
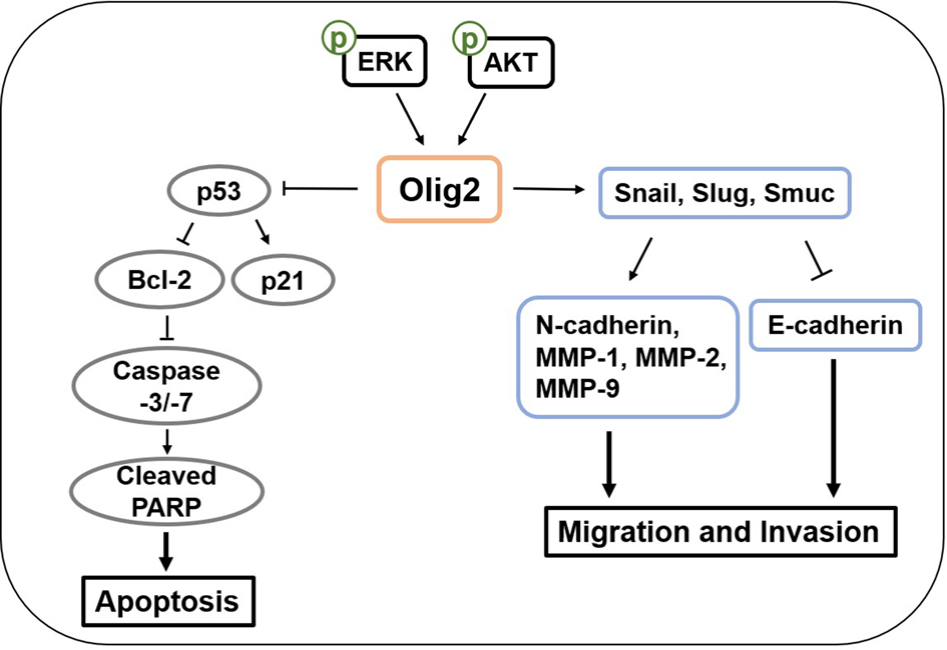
Olig2 regulates apoptosis, migration and invasion of melanoma cells. Both PI3K and MAPK pathways are involved in the regulation of Olig2 in melanoma cells. Inhibition of Olig2 induces p53 apoptosis pathway, then decreases viability of melanoma cells. Olig2 regulates EMT transcription factors such as snail and slug, and subsequently E-cadherin, N-cadherin and MMPs. Down regulation of Olig2 suppressed migration and invasion of melanoma cells.

In conclusion, this study is the first to investigate the molecular mechanism of Olig2 in melanoma cells. We revealed oncogenic roles of Olig2 in melanoma cells by inhibiting p53-mediated apoptosis and promoting migration and invasion of cells through the MAPK and PI3K pathways (Fig. 9). Our findings suggest that Olig2 may be a novel potential regulator of melanoma progression.

## Materials and methods

### Cell culture

A735, 501mel and HM3KO human melanoma cells were cultured in dulbecco's modified eagle's medium (DMEM; Welgene Inc., Korea) supplemented with 10% fetal bovine serum (FBS) (Gibco, Los Angeles, CA, USA) and 1% penicillin/streptomycin. MNT1 human melanoma cell were cultured in minimum essential media (MEM; Welgene Inc., Korea) supplemented with 20% FBS, 1% penicillin/streptomycin and 20 mM 4-(2-Hydroxyethyl)-1- piperazineethanesulfonic acid (HEPES). Normal human melanocyte (NHEM) cells were cultured in Medium 254 (M-254–500; Invitrogen Life Technologies, Carlsbad, CA, USA) containing Human Melanocyte Growth Supplement (HMGS; Invitrogen Life Technologies, Carlsbad, CA, USA) and 1% penicillin/streptomycin at 37 °C in a humidified atmosphere of 5% CO_2_.

### Transfection of cells

For knockdown of Olig2, human melanoma cells were transfected with OLIG2 CRISPR/Cas9 KO Plasmid (h2) (Santacruz Biotechnology, Dallas, TX, USA) using UltraCruz Transfection Reagent (Santacruz Biotechnology, Dallas, TX, USA) for 24 h. Control CRISPR/Cas9 Plasmid (Santacruz Biotechnology, Dallas, TX, USA) was used for negative control.

### Ez-CyToX cell viability assay

Cell viability was examined by Ez-Cytox assay kit (Daeil Lab Service Co Ltd, Seoul, Korea). Cells were seeded at 1 × 10^4^ cells/well in a 96-well plate. Cell viability was detected at 24 h, 48 h and 72 h after transfection of CRISPR-Olig2. 10 μl of Ez-Cytox solution was added to each well and 96-well plates were incubated for an additional 1 h at 37 °C in a 5% CO_2_ incubator. Cell viability was detected at 490 nm by ELISA reader (Tecan, Mannedorf, Switzerland). Relative cytotoxicity was measured by expressing the viability of the cells as a percentage.

### Cell counting assay

Cells were trypsinized after 72 h of transfection of CRISPR/Cas9 plasmid. Equally mix 10 μl of trypan blue dye (Sigma Aldrich, St. Louis, MO, USA) with the cell suspension. After staining cells with trypan blue, the cell number was counted using the hemocytometer. Each measurement was repeated in triplicate.

### Apoptosis assay

Cell apoptosis was analyzed 24 h after transfection using RealTime-Glo Annexin V Apoptosis Assay (Promega Corporation). Cells were seeded in white 96-well polystyrene microplate (Corning, Inc., Corning, NY, USA). Detection reagent was treated according to the manufacturer's instructions. Annexin V-based luminescence was measured using ELISA reader (Tecan, Mannedorf, Switzerland). The fluorescence intensity of each sample is expressed as a percentage.

### Caspase-Glo-3/-7 assay

Activities of caspases-3/-7 were measured using the Caspase-Glo-3/-7 assay (Promega G8091, Whitehead Scientific, Bracken fell, South Africa) in A375 and 501mel cells. Cells were seeded at 1 × 10^4^ cells/well in a 96 well black clear flat bottom plates (Greiner CELLSTAR, Frickerhansen, Germany). After 24 h of transfection of CRISPR/Cas9 plasmid, cells were treated with caspase reagent in the equal amount with the culture solution. Fluorescence was measured on ELISA reader (Tecan, Mannedorf, Switzerland) with excitation at 490 nm and emission at 520 nm. The fluorescence intensity of each sample is expressed as a percentage.

### Wound healing assays

Cells were seeded at 1 × 10^5^ cells/well in 12-well plates. When the cells reach about 90% confluence after transfection of the CRISPR/Cas9 plasmid into the cells, gently scratched the monolayer with a yellow micropipette tip across the center of the each well and washed twice with phosphate buffered saline (PBS). The wound closure was observed at the indicated times. Images were taken with a microscope. All experiments were repeated three times. The wound gap area was quantified by ImageJ software 1.45 s (U.S. National Institutes of Health, Bethesda, MD, USA). Measurement of the aera was calculated by subtracting the area value of 24 h and 48 h from the area value of 0 h and converting the calculated value as a percentage of the control group.

### Transwell invasion and migration assay

For invasion assay, thaw Matrigel (Corning Inc., Kaiserslautern, Germany) on ice in 4 °C refrigerator overnight. Dilute Matrigel in serum free DMEM to final concentration of 0.5 mg/ml. Load 70 μl of Matrigel into the Transwell chamber with 8.0-µm pore size polycarbonate membranes (Corning Inc, Corning, NY, USA) and solidified in a 37 °C incubator. After 24 h, add 200 μl cell suspension (5 × 10^4^ cells/ml in serum freem DMEM) onto Matrigel-coated cell culture insert and add 550 μl DMEM complete media containing 10% FBS into lower chamber. After 24 h, remove the media and Matrigel in upper chamber and wash twice by PBS. Fix cells with 4% formaldehyde for 1 min at room temperature. Remove formaldehyde and wash twice with PBS. And then the invaded cells were stained with 4′,6-diamidino-2-phenylindole (DAPI) for visualization of the nuclei. The invasive cells were detected by fluorescence microscope at × 100 magnification. The stained nuclei were counted and quantified using ImageJ software 1.45 s (U.S. National Institutes of Health, Bethesda, MD, USA).

For migration assay, load 200 μl cell suspension (5 × 10^4^ cells/ml in serum freem DMEM) onto Transwell chamber with 8.0-µm pore size polycarbonate membranes and add 550 μl DMEM complete media containing 10% FBS into lower chamber. After 18 h, remove the media and wash twice by PBS. Fix cells with 4% formaldehyde for 1 min at room temperature. Remove formaldehyde and wash twice with PBS. And then the invaded cells were stained with DAPI for visualization of the nuclei. The migrated cells were detected by fluorescence microscope at × 100 magnification. The stained nuclei were counted and quantified using ImageJ software 1.45 s (U.S. National Institutes of Health, Bethesda, MD, USA).

### Gelatin zymography

The gelatinolytic activities of MMP-2/-9 were assessed by gelatin zymography assay. 24 h after transfection of 2 μg of CRISPR/Cas9 plasmid, the conditioned media were collected and centrifuged for concentration using the 50 K Amicon Ultra Centrifugal Filters. Filtered media was assayed by electrophoresis on 10% zymogram gel (Novex 10% Zymogram Plus (Gelatin) Protein Gels, Thermo Fisher Scientific, MA, USA) with 1 mg/ml gelatin. The gel was run at 120 V for 1.5 h at 4 °C. Novex Tris–Glycine SDS Running Buffer (10X) was used as electrophoresis reagent. After electrophoresis, incubate the gel with renaturing buffer (Novex Zymogram Renaturing Buffer (10X), Thermo Fisher Scientific, MA, USA) under gentle agitation for 30 min. Replace with developing buffer (Novex Zymogram Developing Buffer (10X), Thermo Fisher Scientific, MA, USA) and incubate the gel at 37 °C overnight. After washing with distilled water three times, the gels were stained with SimplyBlue safe stain (Thermo Fisher Scientific, MA, USA) overnight under gentle agitation at room temperature. The gel was de-stained with solution of 10% acetic acid, 40% methanol and 50% distilled water to visualize the zymogen bands. The zymography gels were scanned and analyzed using the ImageJ software 1.45 s (U.S. National Institutes of Health, Bethesda, MD, USA).

### MMP-1 ELISA assay

Cells were seeded at 3 × 10^4^ cells/well in 48-well plates. 24 h after transfection of the CRISPR/Cas9 plasmid, cell culture supernatants were collected and stored at − 20 °C. A human MMP-1 ELISA Kit (R&D Systems GmbH. Wiesbaden, Germany) were used to measure the levels of MMP-1 according to the manufacturer’s instructions.

### Reverse transcription polymerase chain reaction (RT-PCR) assays

Total RNA was isolated using TRIZOL reagent (Takara Bio, Inc., Tokyo, Japan) according to the manufacturer's instruction. Complementary DNA (cDNA) was synthesized by reverse transcriptase reaction. PCR amplification was performed with specific primers using thermal cycler SimpliAmp, Thermo Fisher Scientific, MA, USA). The products were run on 2% agarose gel (Bioneer Co., Daejeon, Korea) in 1X Tris Acetate-EDTA (TAE) buffer (Elpis Biotech, Daejeon, Korea) containing RedSafe Nucleic Acid Staining Solution (iNtRON Biotechnology, Seongnam, Korea). Sequences of primers used for RT-PCR: Olig2 forward 5′-GAATCCGCTGGTATCCACGA-3′, Olig2 reverse 5′-GCGGAGCGAGACGTTTAGAA-3′, MMP-2 forward 5′-GGCCCTGTCACTCCTGAGAT-3′, MMP-2 reverse 5′-GGCATCCAGGTTATCGGGGA-3′, MMP-9 forward 5′-GATGCGTGGAGAGTCGAAAT-3′, MMP-9 reverse 5′- CACCAAACTGGATGACGATG-3′, β-actin forward 5′-GGCATCGTGATGGACTCCG-3′, β-actin reverse 5′- GCTGGAAGGTGGACAGCGA-3’.

### Antibodies

Antibodies to detect p53 (DO-1), Slug (A-7) and Smuc (E-3) were from Santacruz Biotechnology. Olig2 (H-10) antibody was from Santacruz Biotechnology and Olig2(AF2418) antibody was from R&D systems. Antibodies against p42/44 MAPK (ERK1/2), phospho-42/44 MAPK (ERK1/2), PARP, Bcl2, Snail (C15D3), E-cadherin (24E10), N-cadherin (D4R1H), phospho-AKT (s475), AKT and p21^*Waf1/Cip1*^ (12D1) were from Cell Signaling.

### Western blot analysis

To gain whole cell lysate, cells were lysed with Radio-Immune Precipitation Assay buffer (RIPA buffer) (Noble Bio, Hwaseong, Korea) containing protease inhibitor cocktail (Sigma, St. Louis, MO, USA) and 1 mM PMSF. Cell lysate was normalized for protein concentration using a BCA assay. For immunoblotting, each protein sample was mixed with NuPAGE LDS Sample and NuPAGE sample reducing agent (Thermo Fisher Scientific, Carlsbad, CA, USA) and heat at 95 °C for 10 min. Samples are loaded onto NuPAGE 4–12% Bis–Tris Protein Gels (Invitrogen ThermoFisher Scientific, Carlsbad, CA, USA). The gel was run at 200 V room temperature and transferred to a polyvinylidene difluoride transfer membrane (PVDF membrane, PALL Corporation, Port Washington, NY) at 4 °C for 2 h. The blots were cut prior to incubate with primary antibodies and the membranes were blocked with 5% bovine serum albumin (BSA) in Tris Buffered Saline (TBS) containing 10 mM Tris–HCl (pH 7.5), 0.1% Tween-20 and 100 mM NaCl for 1 h at room temperature. The membranes were incubated with incubated with primary antibodies at 4 °C for 24 h and then incubated with HRP-conjugated donkey anti-rabbit IgG antibody (Bethyl Laboratories, Montgomery, TX) or goat anti-mouse IgG antibody (Bio-Rad, Hercules, CA) for 1 h at room temperature. After washing, protein bands were detected with the SuperSignal West Pico PLUS Chemiluminescent Substrate and visualized with Fluor Chem E (ProteinSimple, San Jose, CA, USA).

### Meta‑analysis of gene expression profiling

Three microarray studies for melanoma samples were available in NCBI GEO, coded as GSE7553. The gene expression values of Olig2 (Probe set 213824_at) was conducted with the Affymetrix Expression Console software. p-values were calculated using a Student t-test. The gene level of Olig2 was normalized by the level of GAPDH across the samples.

### Immunohistochemical analysis

For immunohistochemistry staining of Olig2, Human melanoma tissue microarray (human TMA) slide (US Biomax, Inc., MD, USA) was initially pre-warmed on a slide warmer (J-HSWD, JISICO, Korea) for 30 min at 60 °C. After cooling the slide at room temperature for at least 1 h, the slide was deparaffinized and gradually rehydrated through descending graded series of 100%, 95% and 70% ethanol and distilled water. After rehydration, antigen retrieval (S1699, DAKO, Carpinteria, CA, USA) was immediately conducted using a high-pressure cooker. After antigen retrieval, the hot slide chamber was cooled on ice for at least 1 h until retrieval solution became clear. To block endogenous peroxidase, the slide was incubated in 3% H_2_O_2_ for 30 min and washed two times with PBS. For blocking non-specific signal, slide was incubated with protein block serum-free (X0909, DAKO, Carpinteria, CA, USA) on humidity chamber. Anti-Olig2 (1:200, ab109186, abcam, Cambridge, Cambs, UK), which was diluted in antibody dilution solution (S1699, DAKO, Carpinteria, CA, USA) was incubated overnight at 4 °C. After three washes in PBS, the slide was incubated in HRP-conjugated anti-rabbit secondary antibody (DAKO, K4003, Carpinteria, CA, USA) for 15 min at room temperature. DAB (K3468, DAKO, Carpinteria, CA, USA) was used for development of antibody, and Mayer’s hematoxylin (S3309, DAKO, Carpinteria, CA, USA) was used for nuclear counterstaining. Positive pixels of Olig2 were counted using QuPath software (developed by University of Edinburgh).

## Supplementary Information

Supplementary Information
