# Supplementary material for: Olig2 regulates p53-mediated apoptosis, migration and invasion of melanoma cells
Source: Sci Rep. 2021 Apr 8;11:7778. doi: 10.1038/s41598-021-87438-x (PMC8032681; doi:10.1038/s41598-021-87438-x)
Supplement: Supplementary file 1 — Supplementary Information [file 41598_2021_87438_MOESM1_ESM.docx]

Supplementary Information

**Olig2 regulates p53-mediated apoptosis, migration and invasion of melanoma cells**

Ji Eun Lee^1^, Sungjin Ahn^2^, Haengdueng Jeong^3^, Seungchan An^2^, Cheol Hwan Myung^1^, Jeong Ah Lee^1^, Sung Chan Hong^1^, Youn Jin Kim ^1^ Jin Young Kim^1^, Jong Hyuk Ryu^1^, Minsoo Noh^2^, Ki Taek Nam^3^, Jae Sung Hwang^1,*^


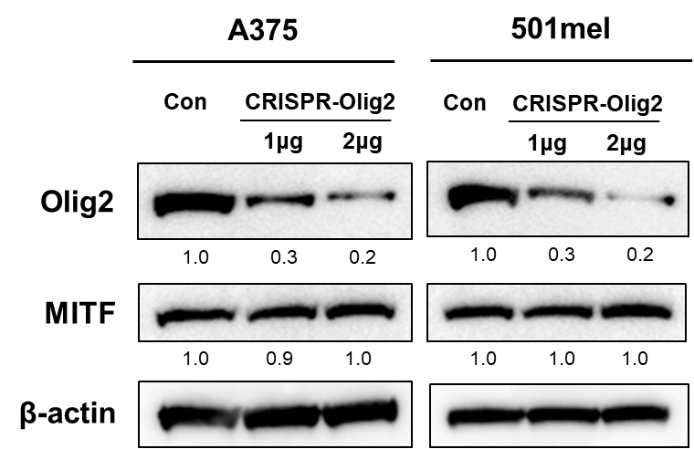


**Supplementary Figure 1. Olig2 knock-down had no effect on MITF expression.**

Protein expression of MITF after silencing of Olig2 expression was detected in A375 and 501mel melanoma cells by western blot analysis. β-actin was used as loading control. The band intensity was quantified using NIH ImageJ software 1.45s.

**(a)**

**
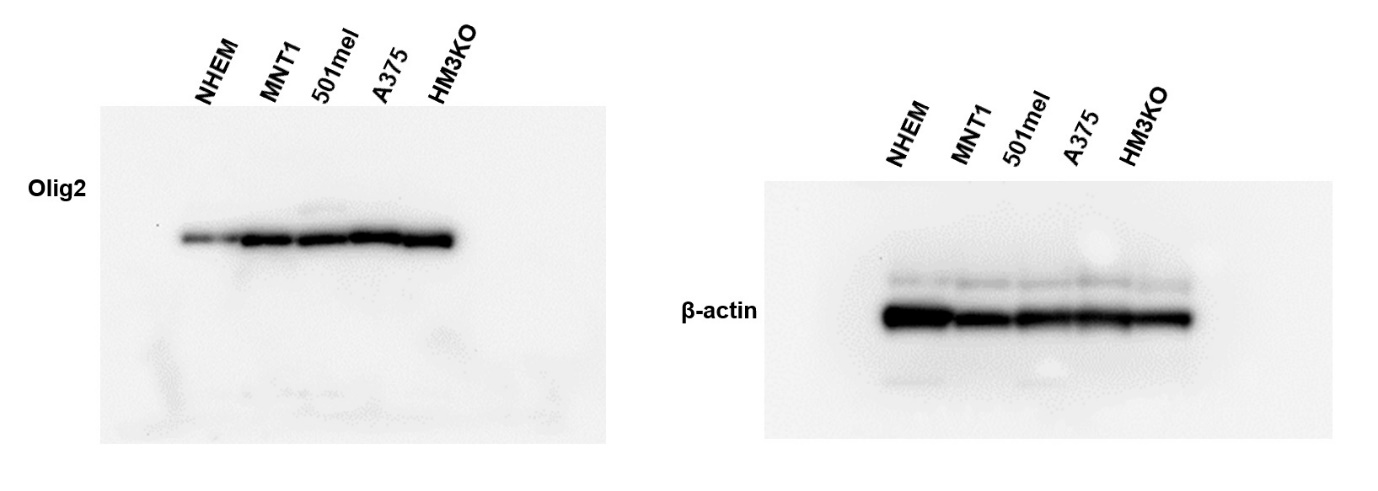
**

**(b)
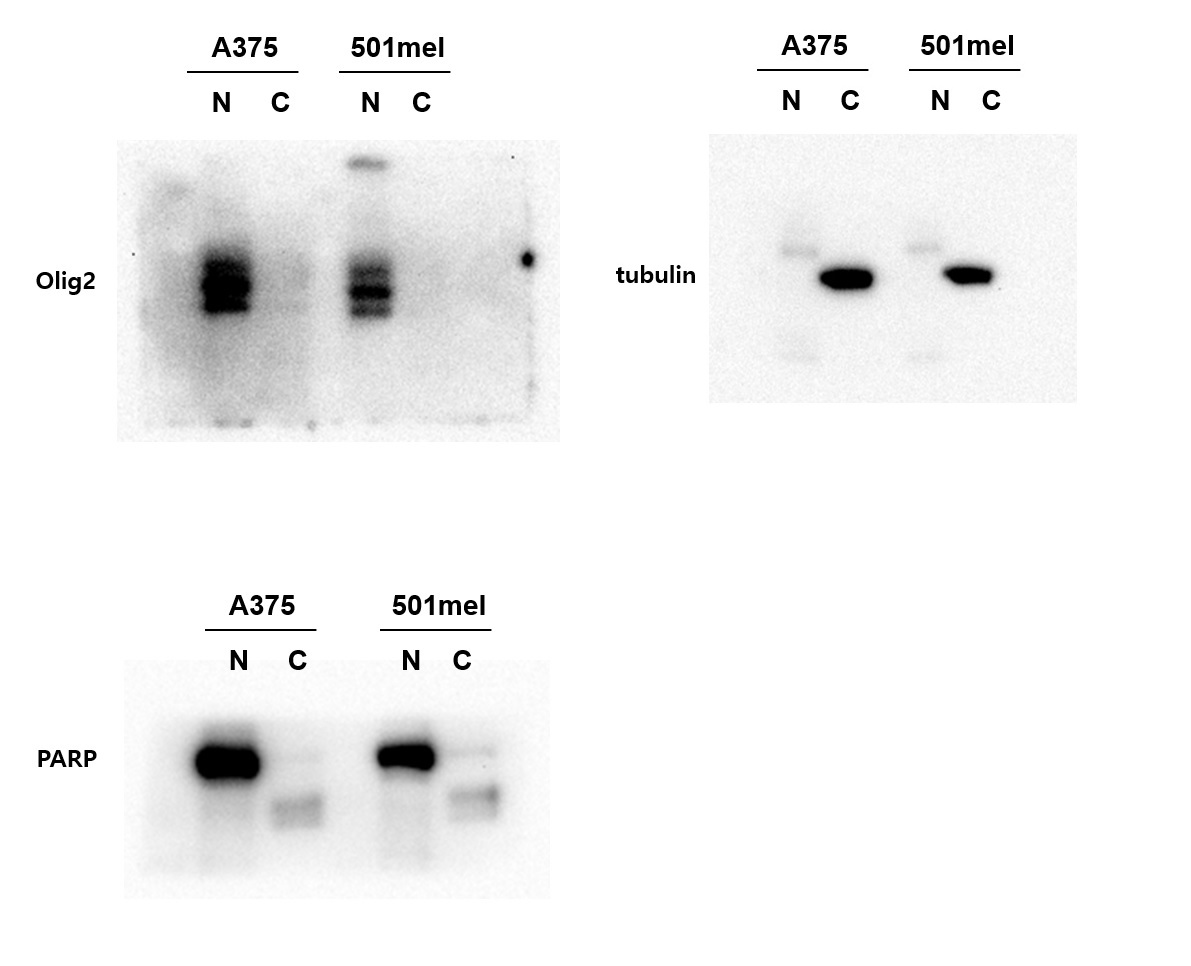
**

**(c)**

**
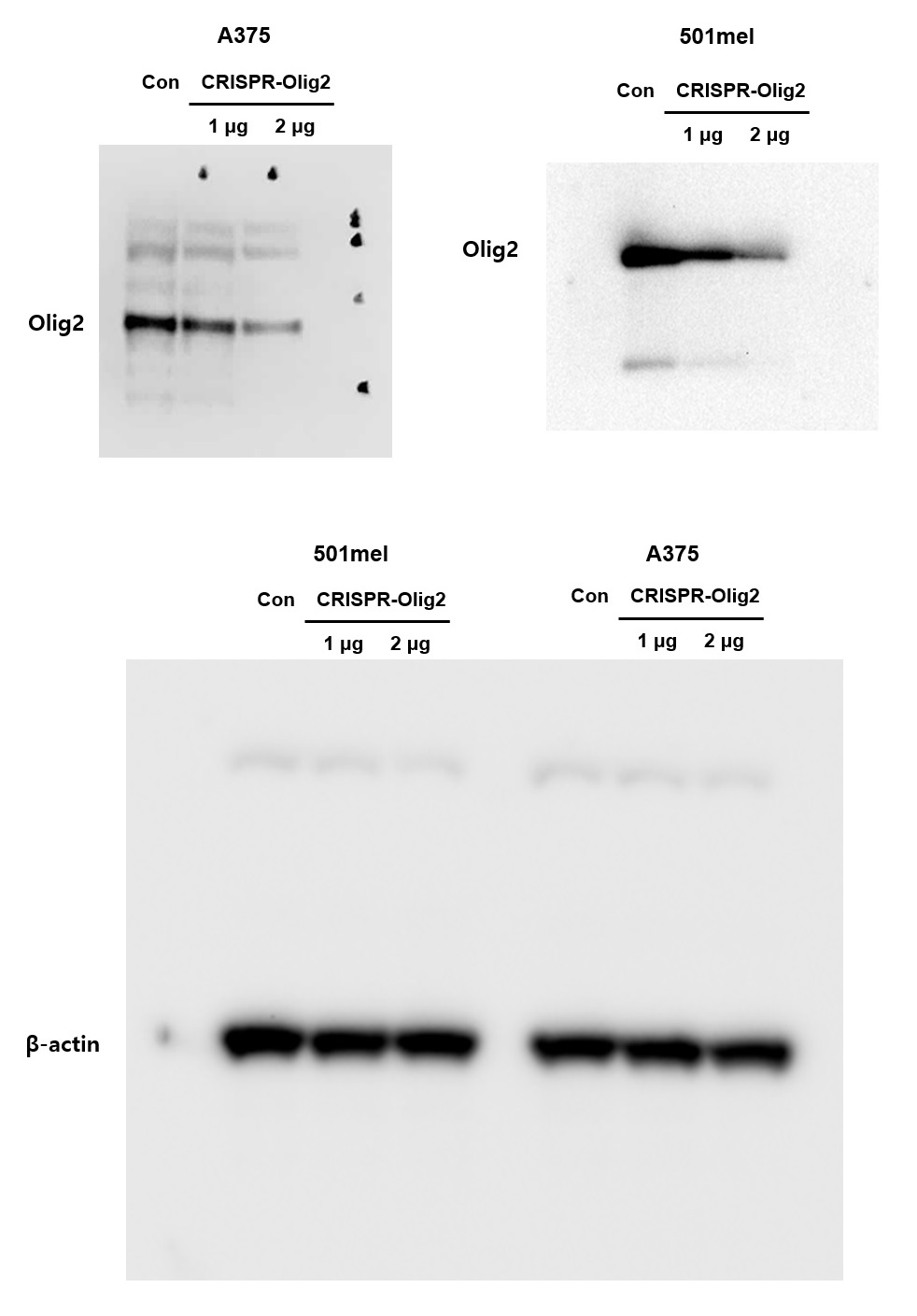
**

**(d)**

**
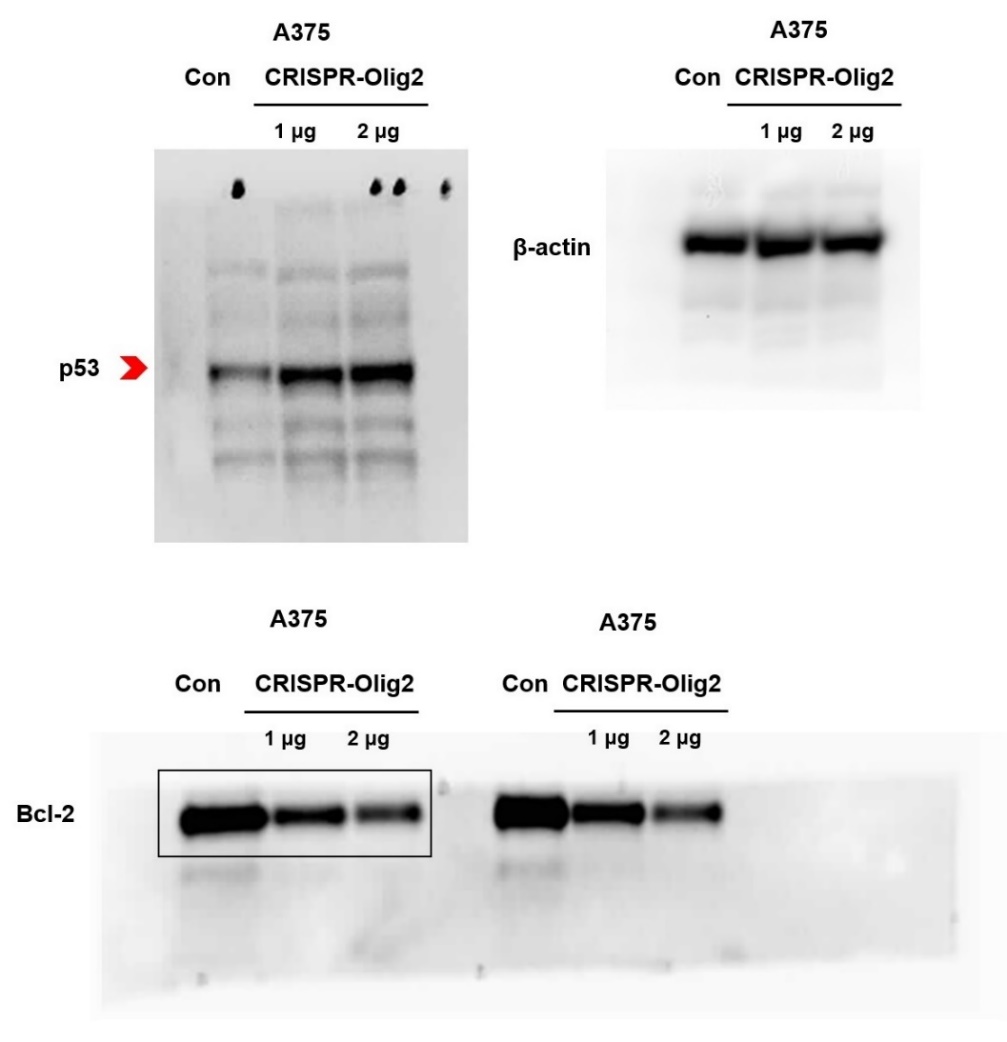
**

**
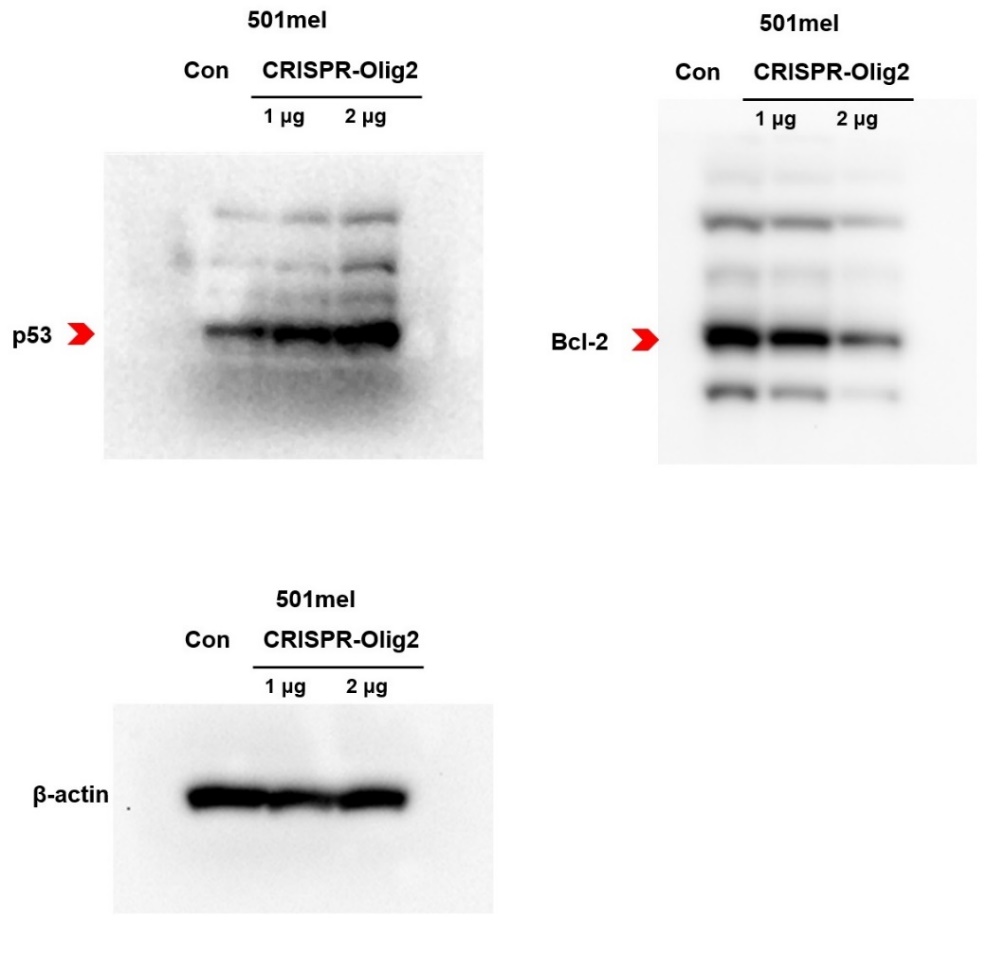
**

**
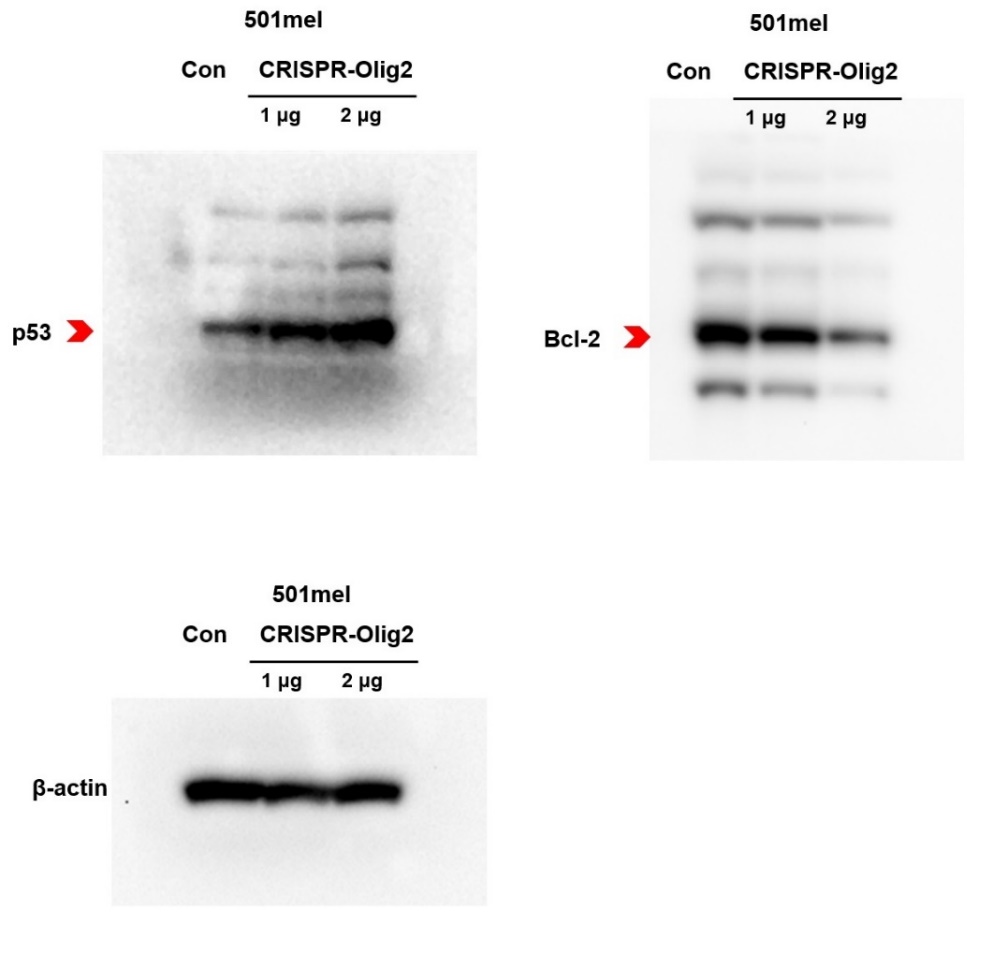
**

**(e)**

**
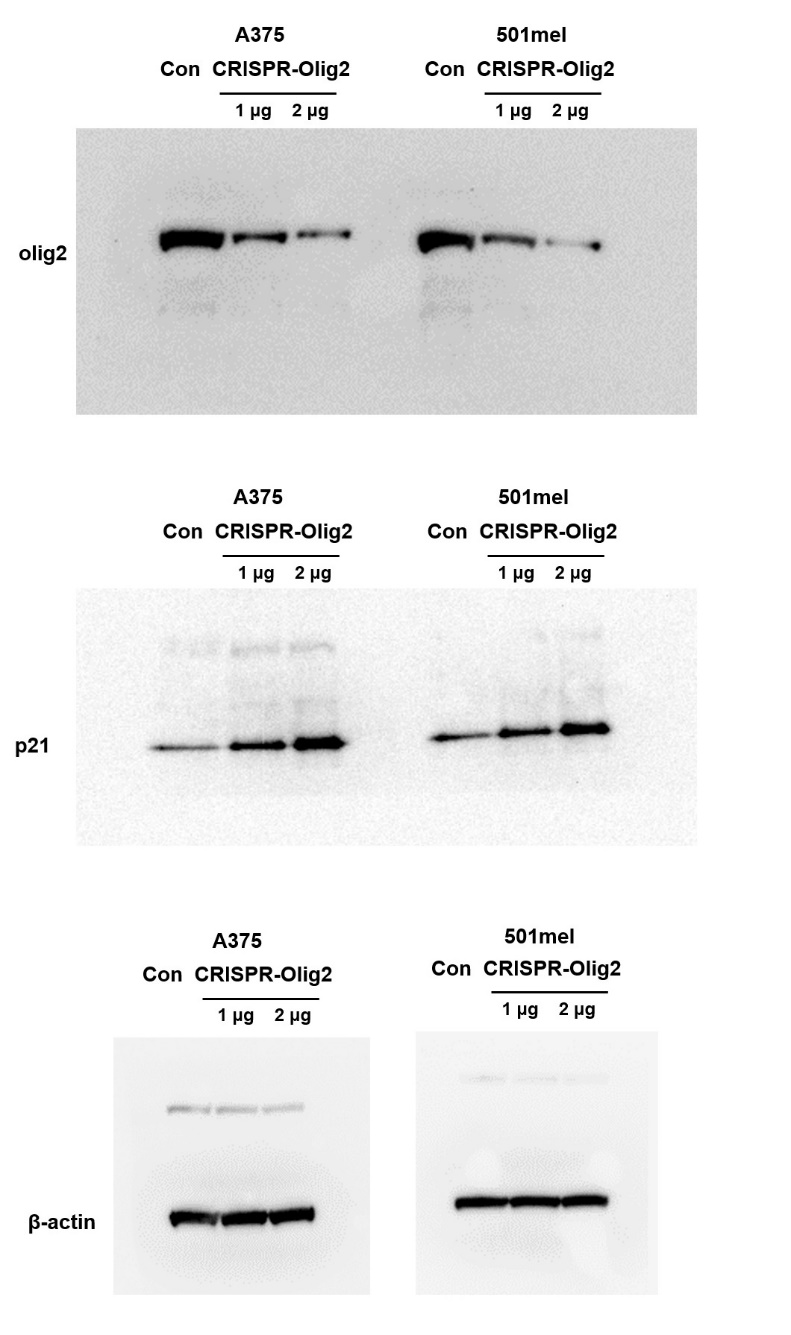
**

**(f)**


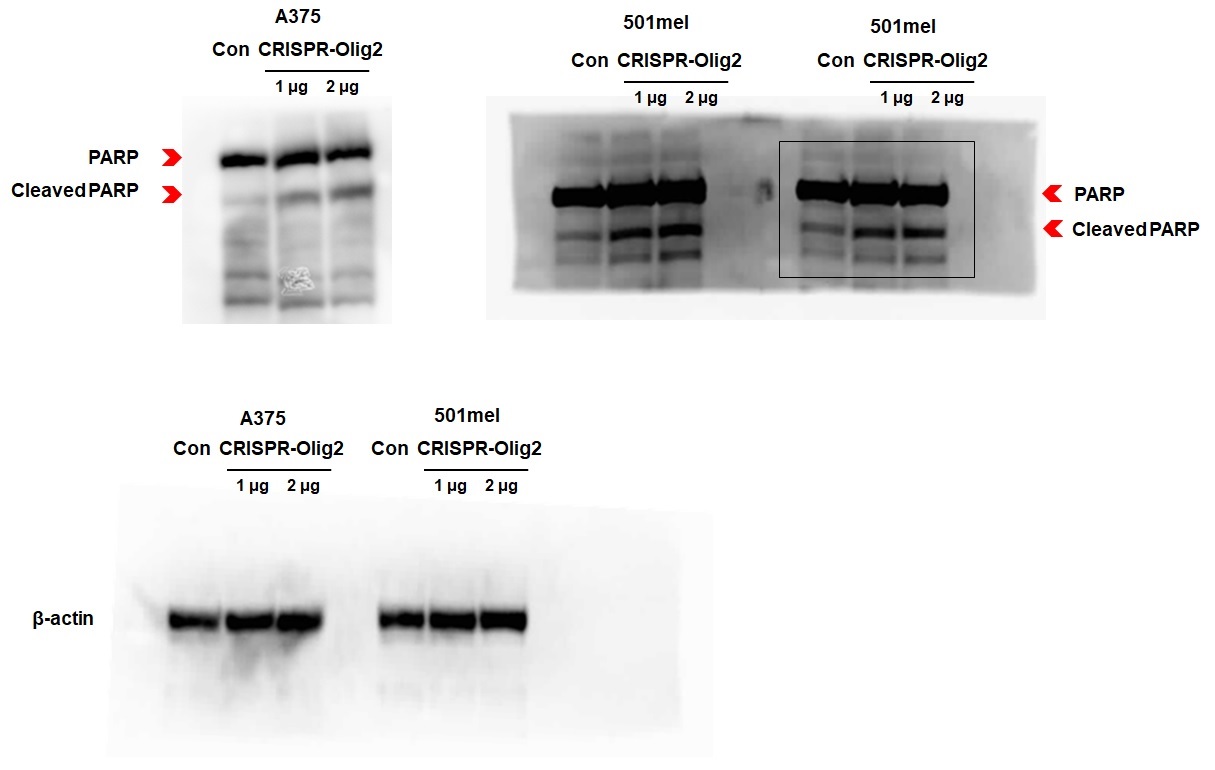


**(g)**

**
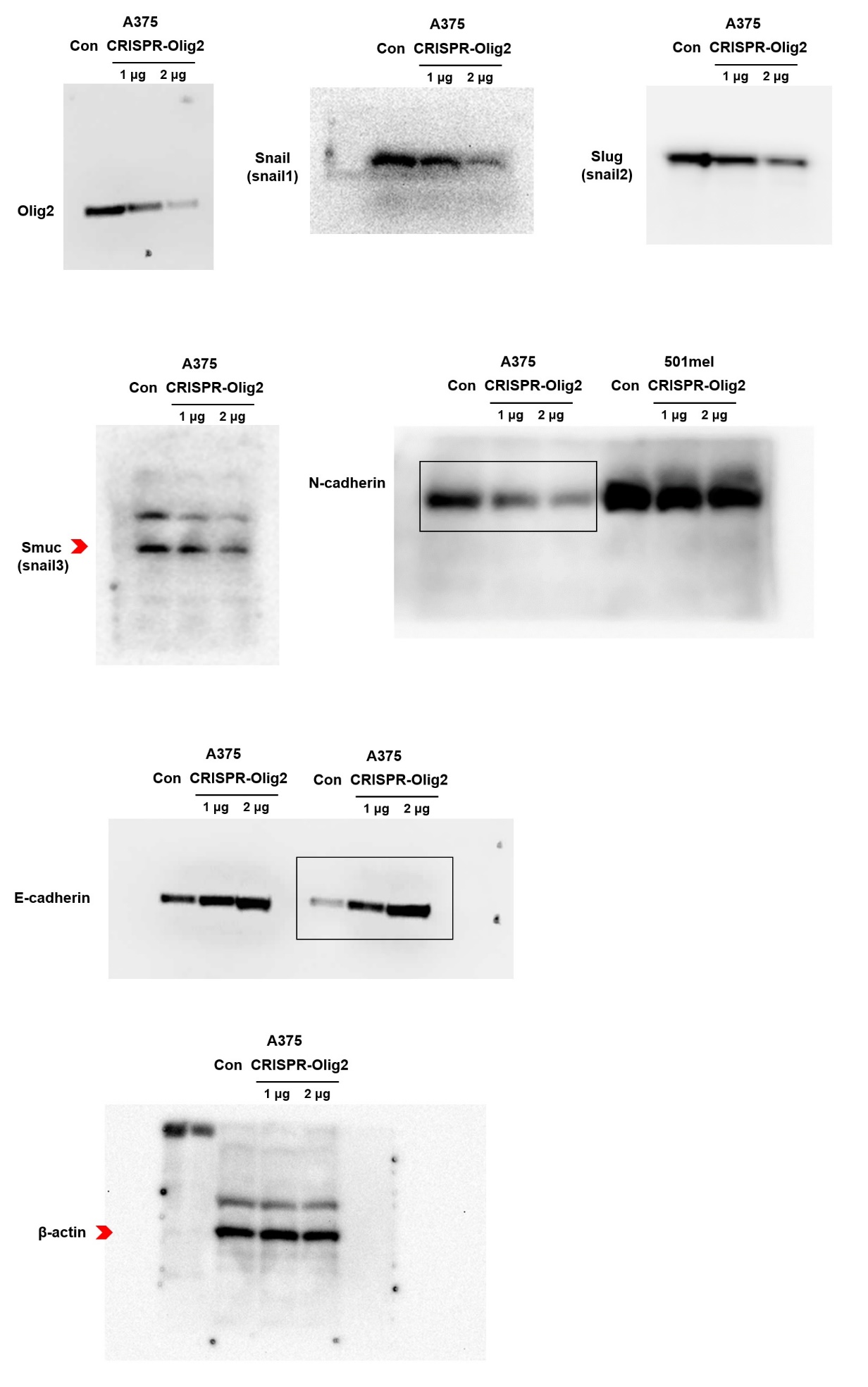
**

**(h)**

**
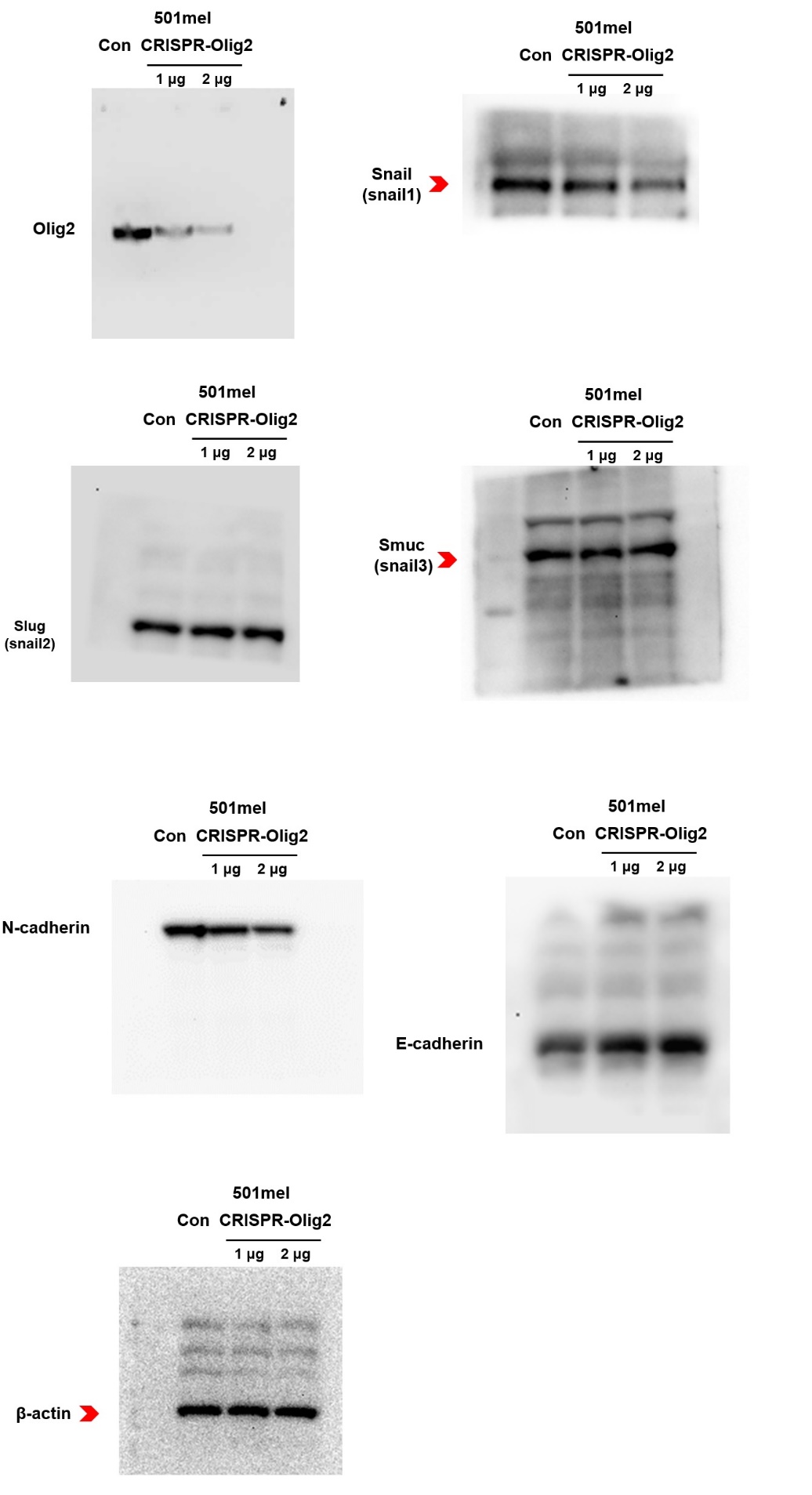
**

**(i)**

**
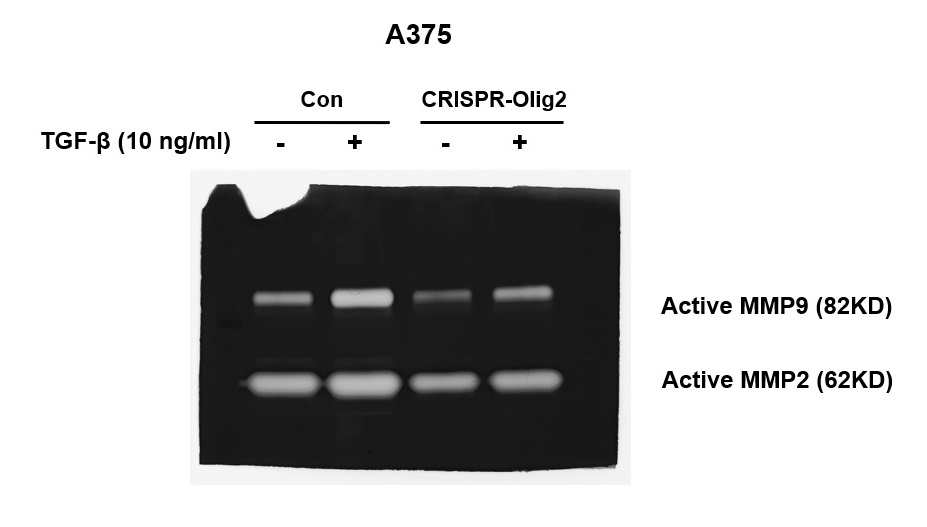
**

**(j)**

**
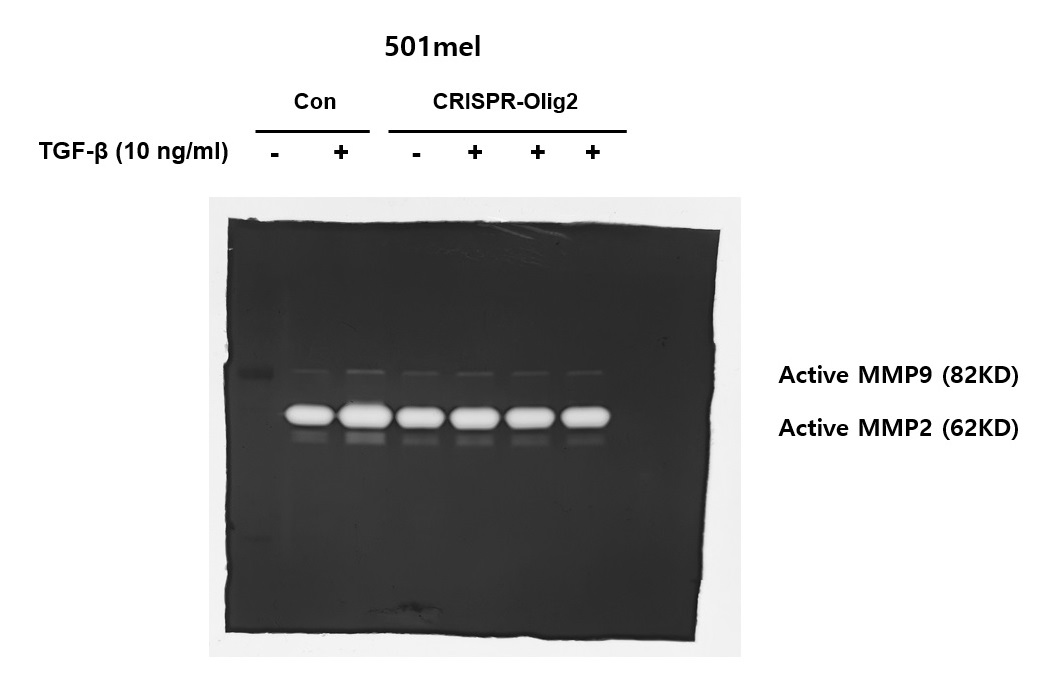
**

**(k)**

**
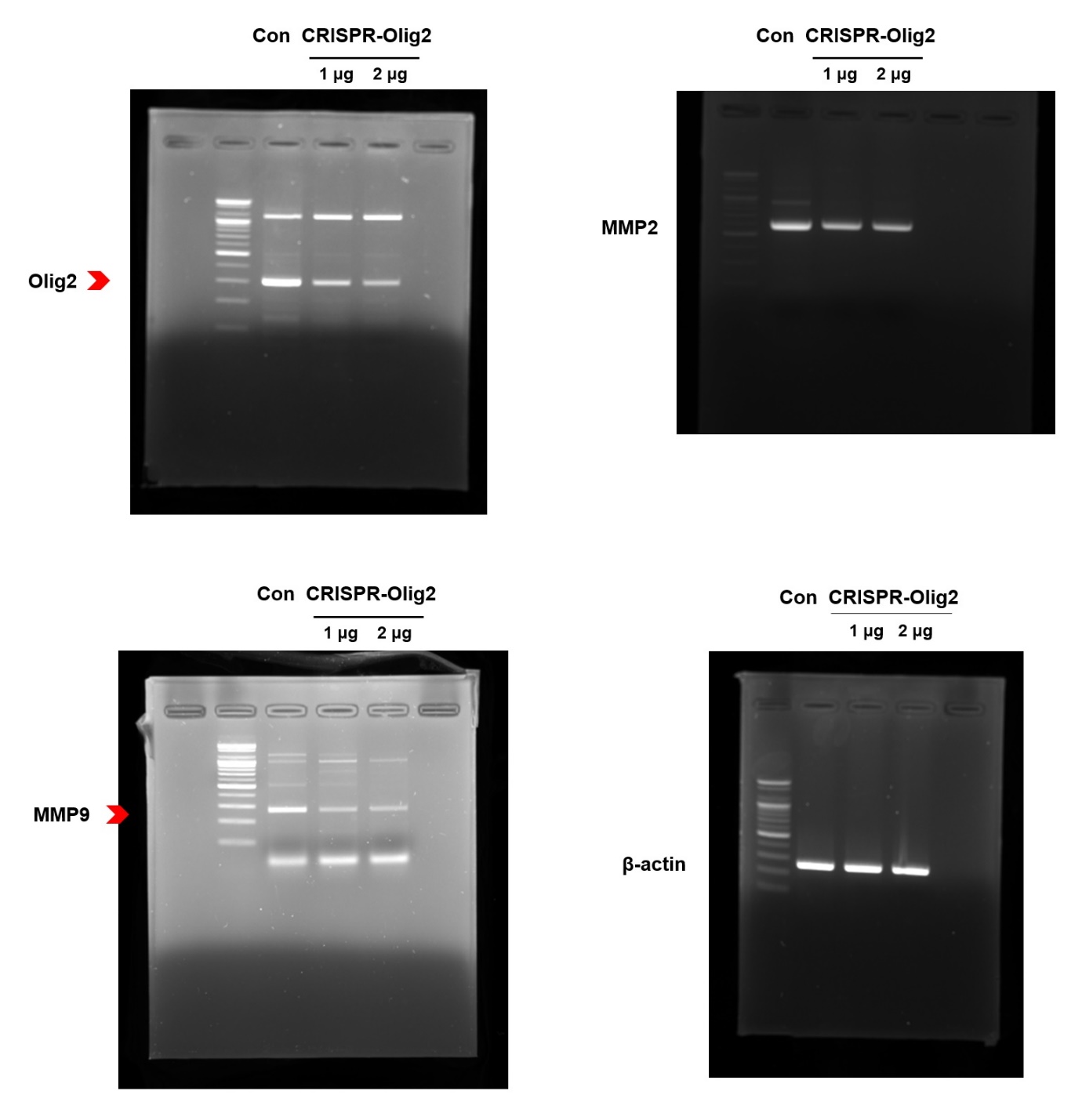
**

**(l)**

**
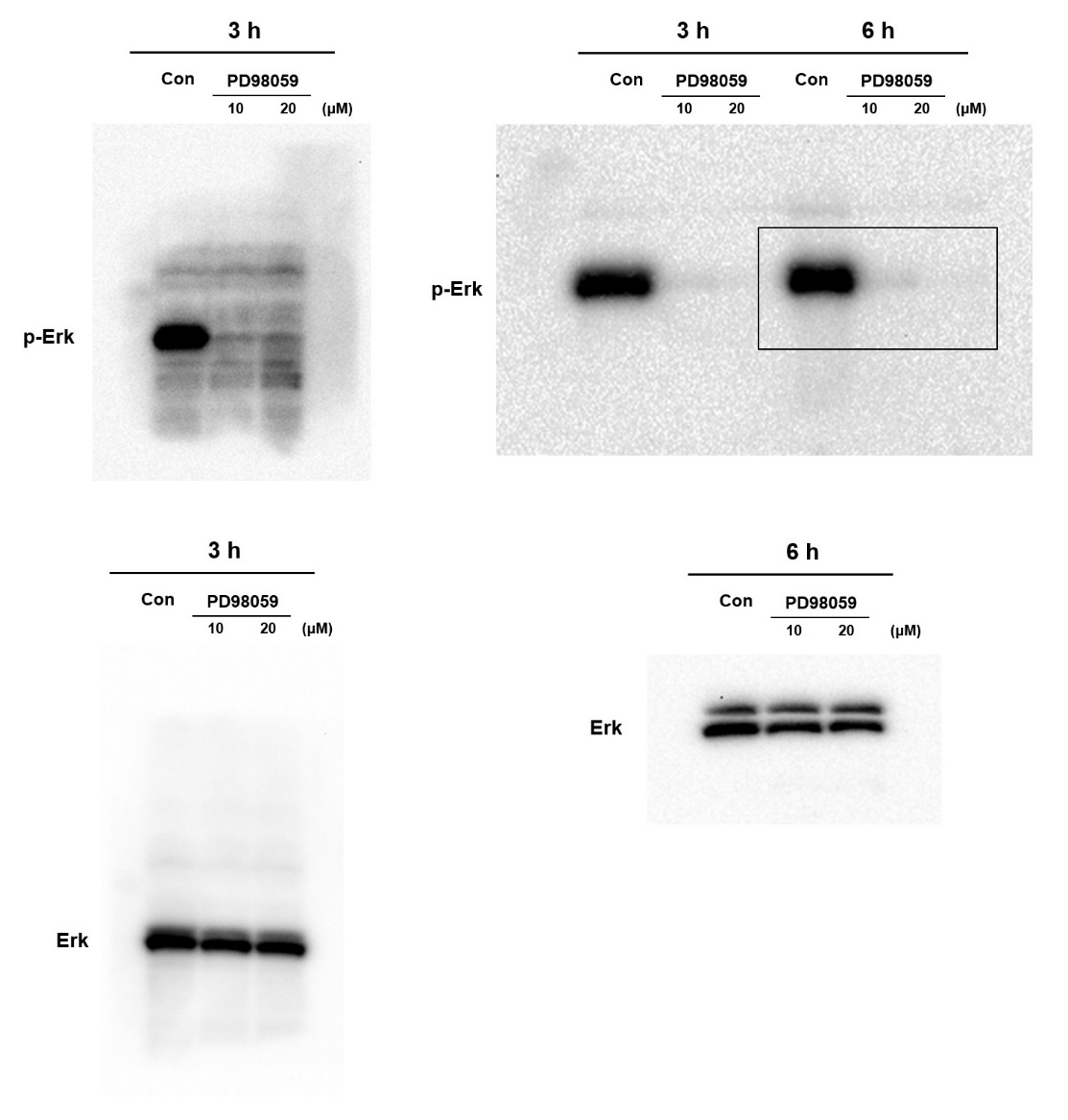
**

**
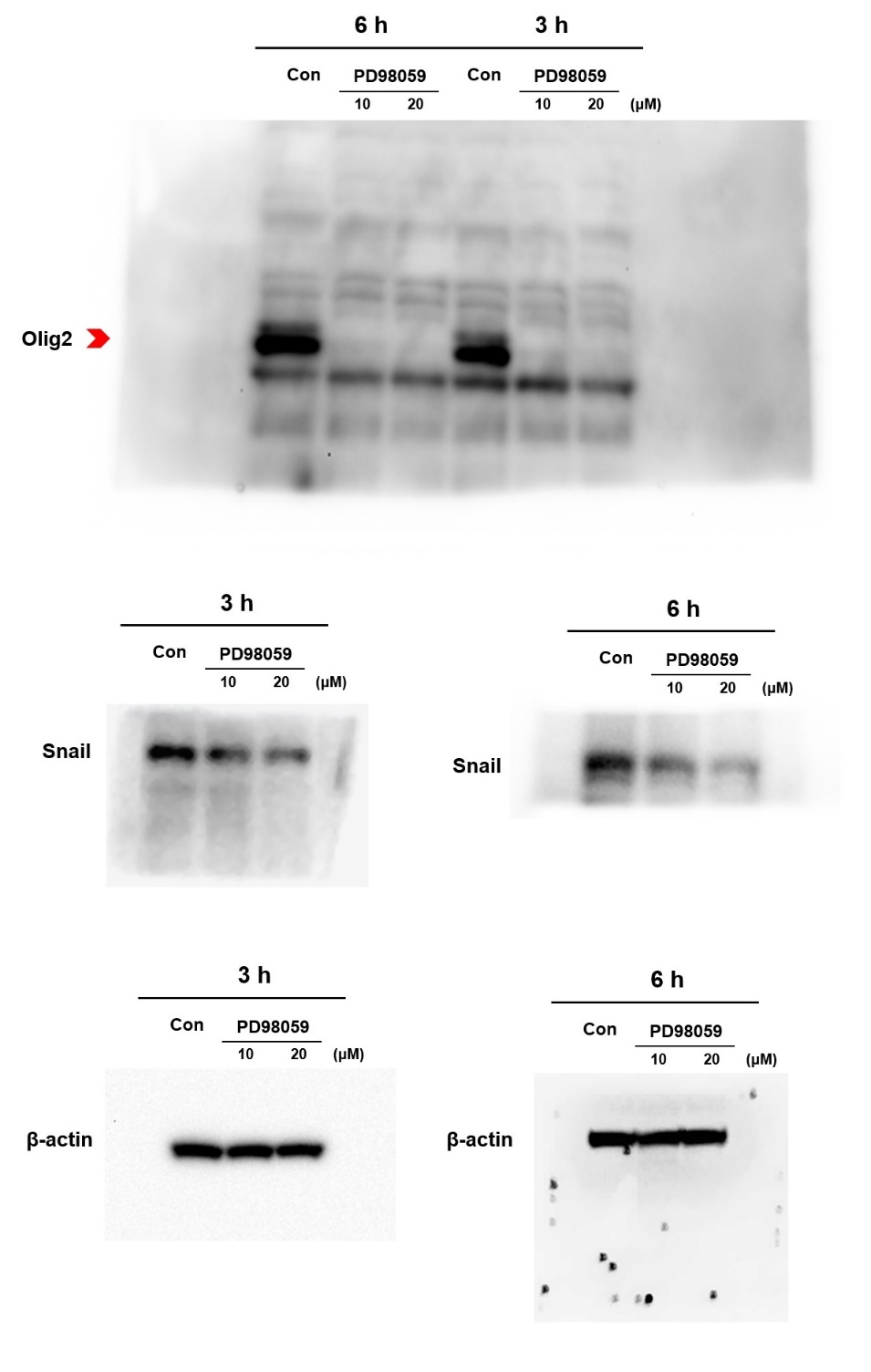
**

**
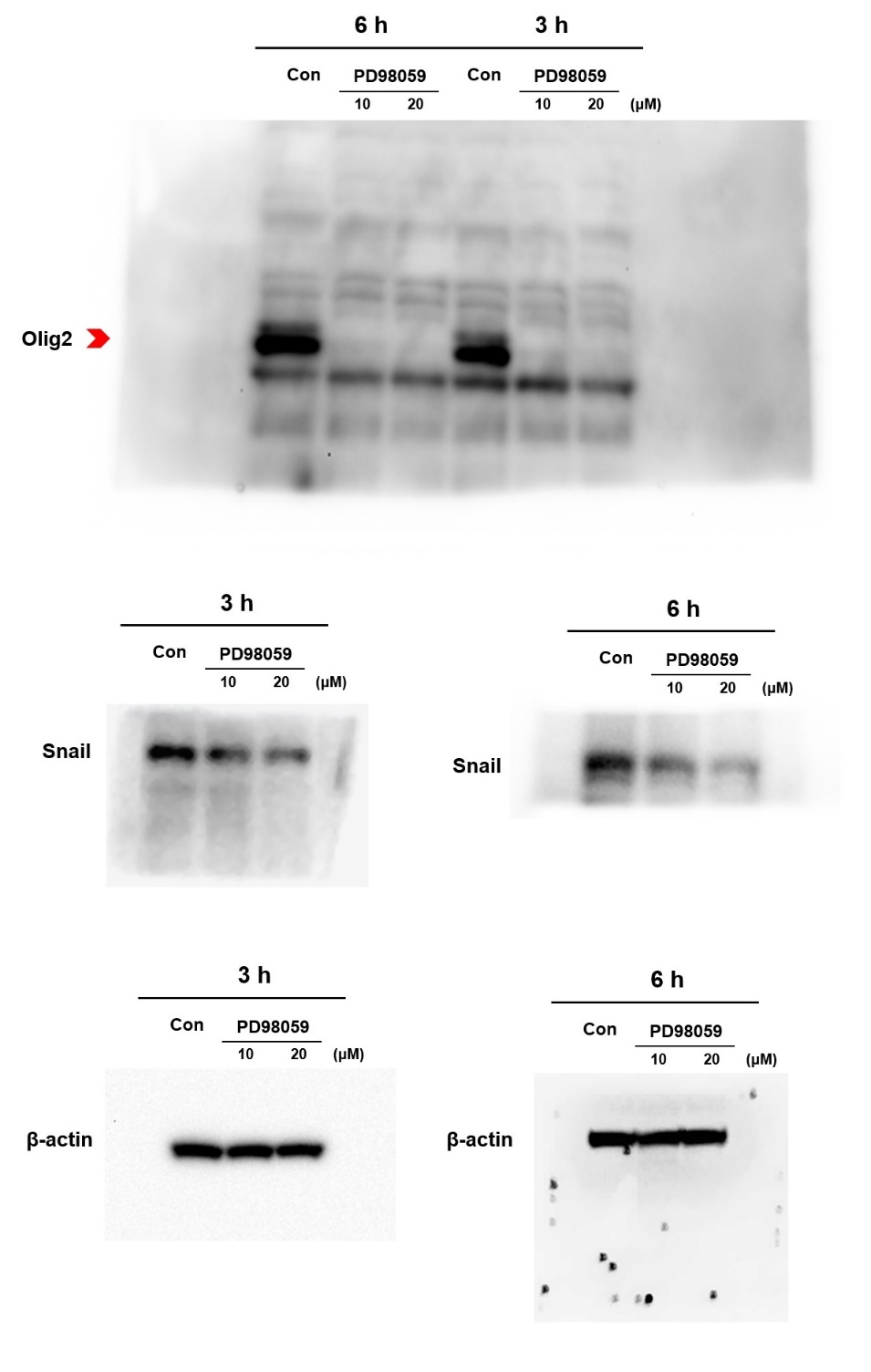
**

**(m)**

**
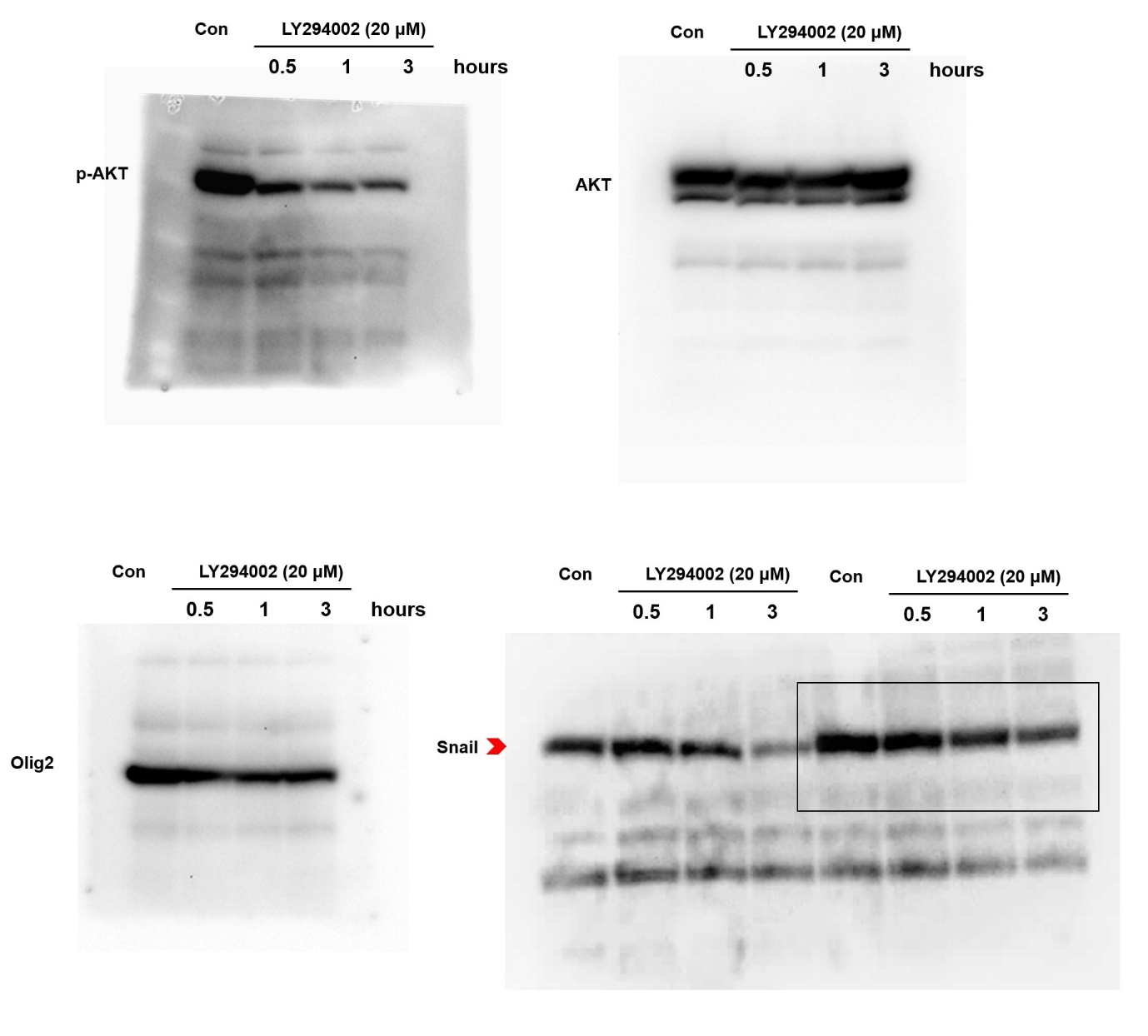
**

**(n)**

**
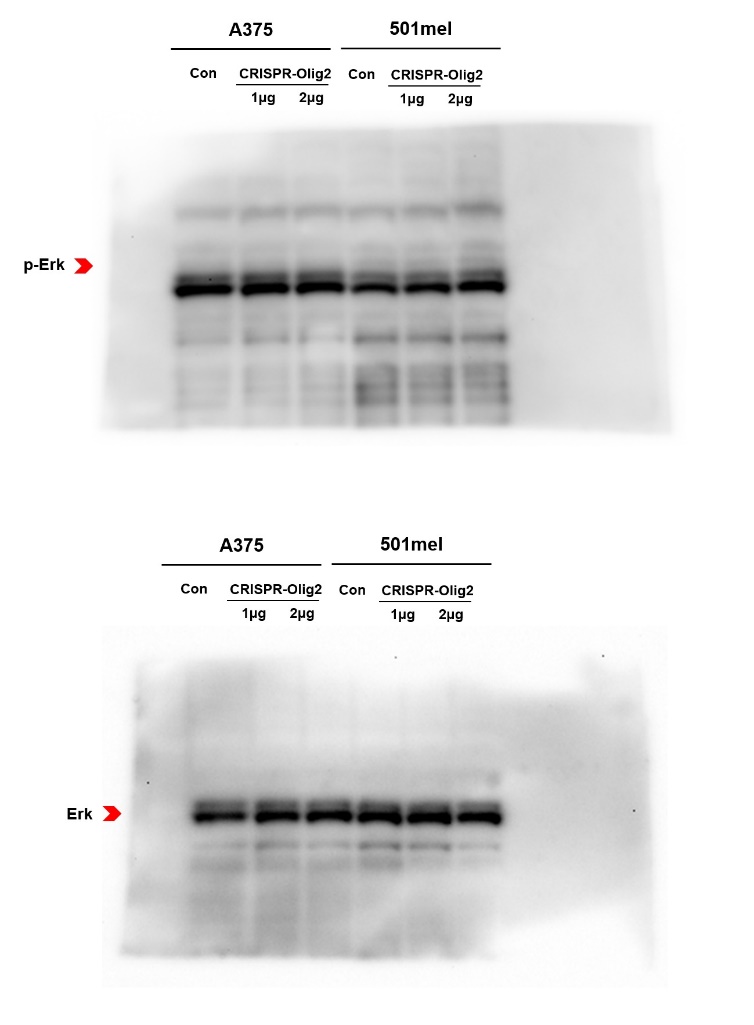
**

**(o)**

**
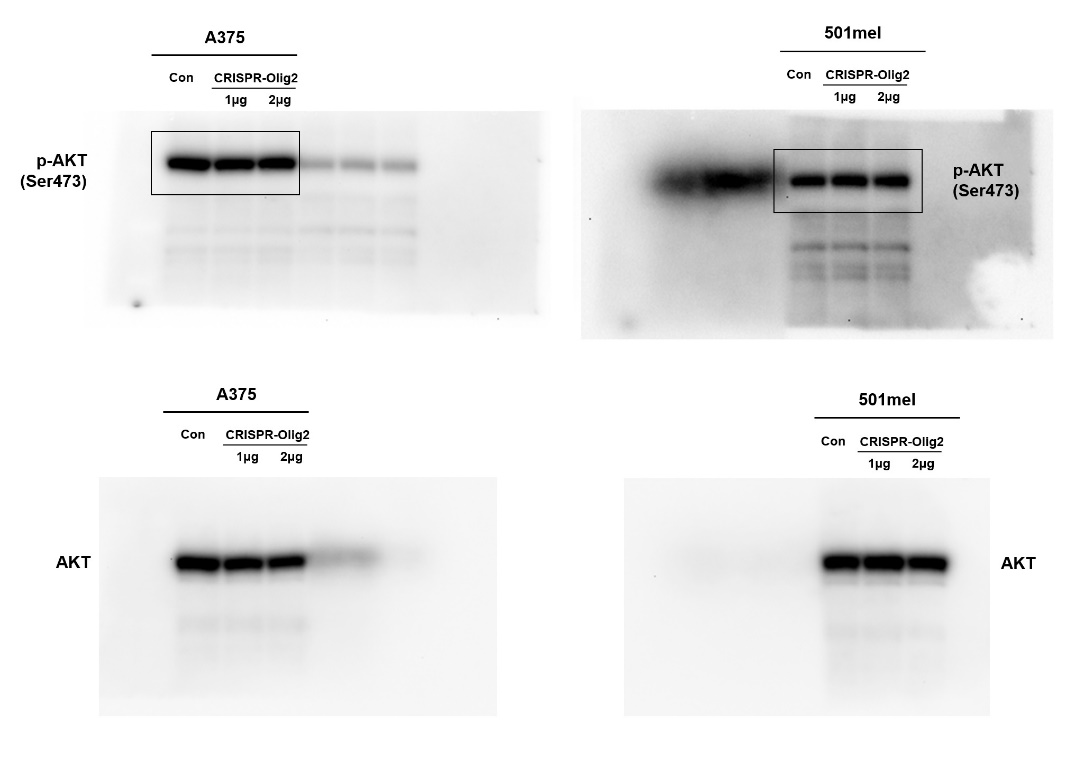
**

**(p)
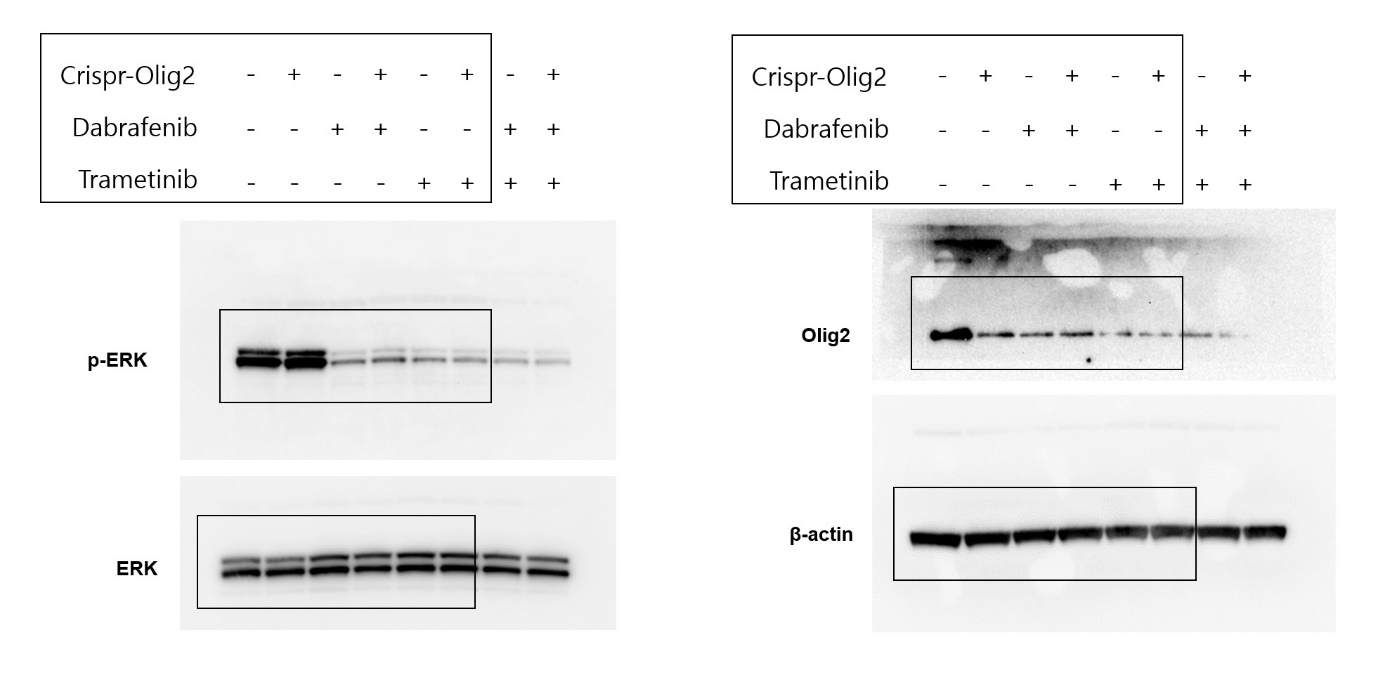
**

**(q)
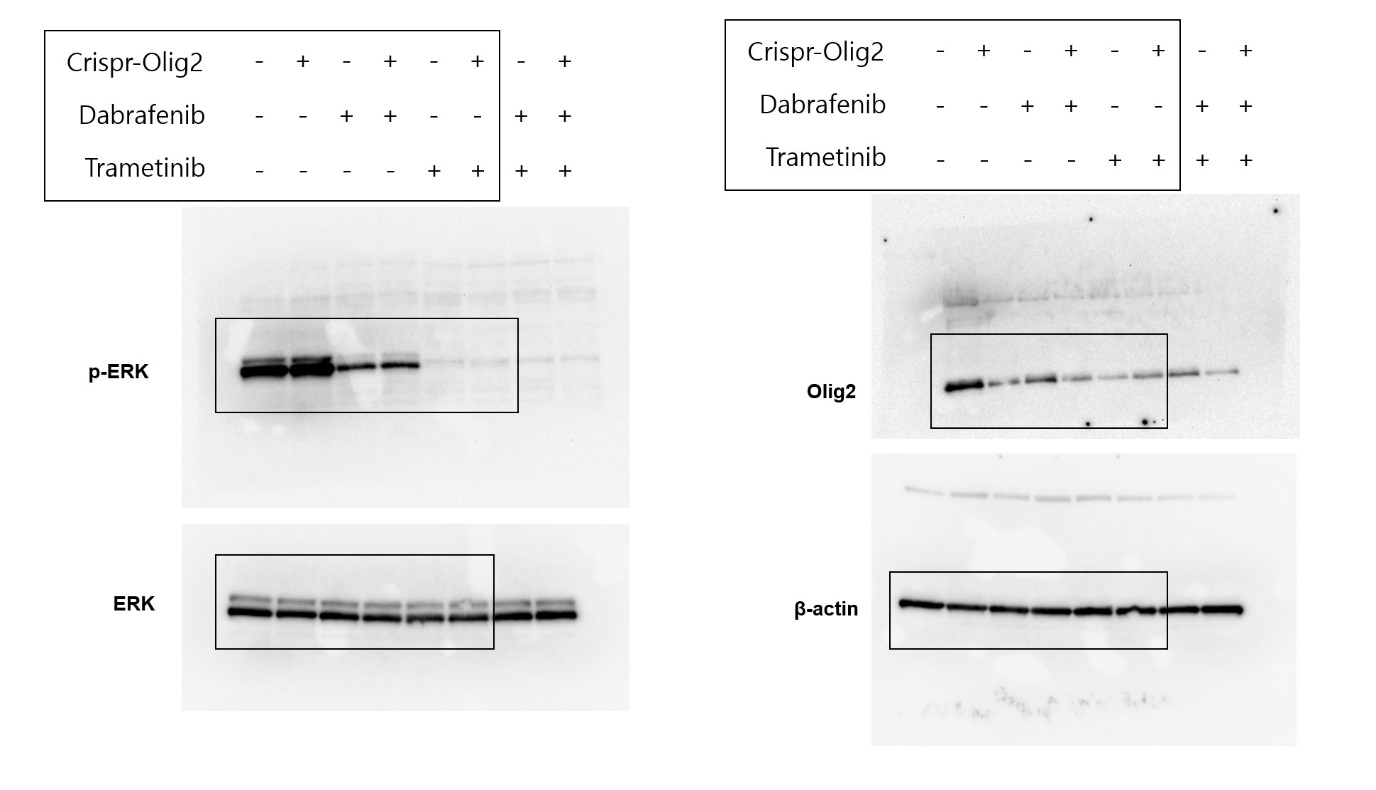
**

**(r)**

**
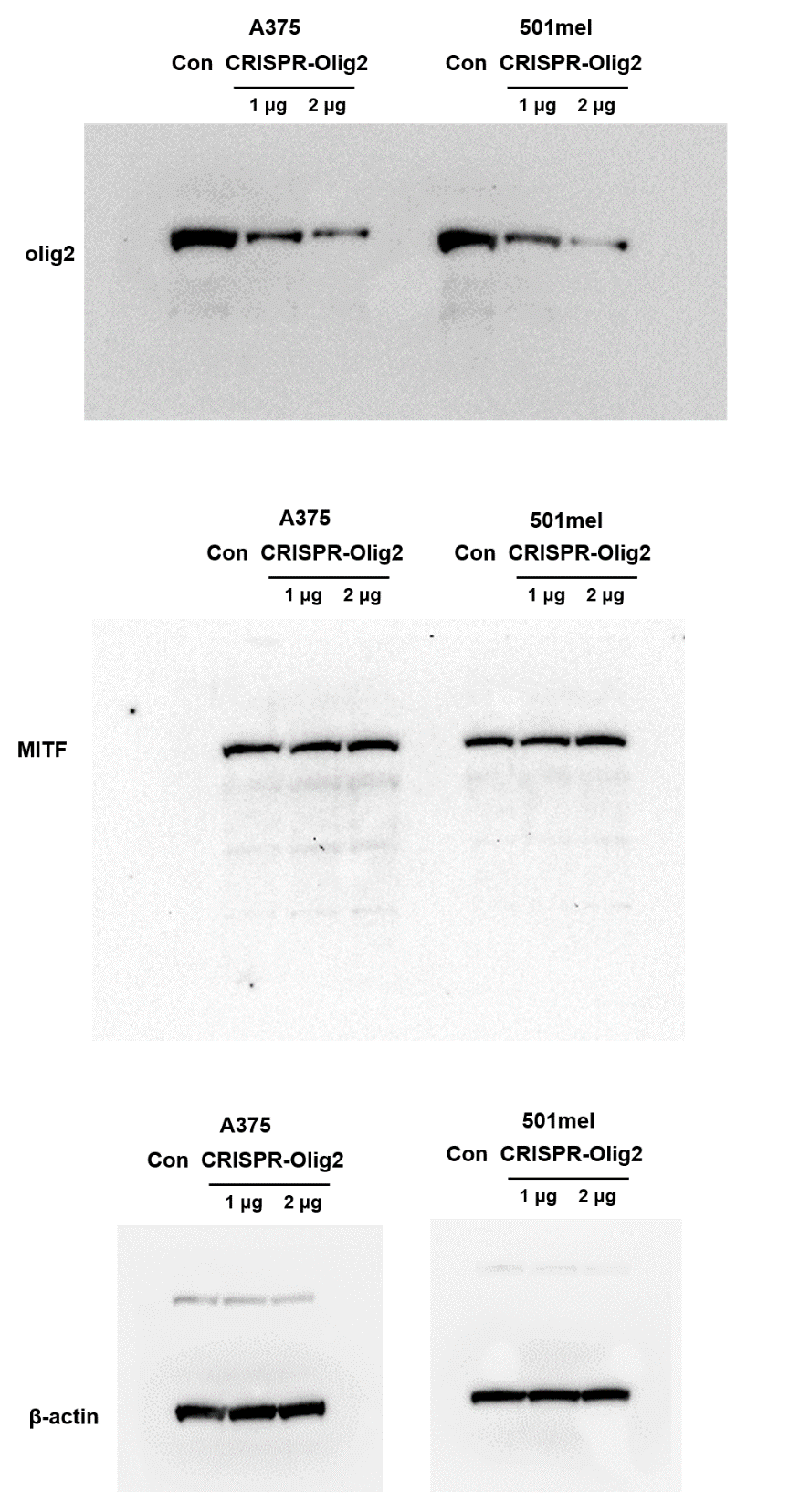
**

**Supplementary Figure 2.**  **Full-length western blot and gel images**.

(a) Full-length blot image for figure 1d. (b) Full-length blot image for figure 2a. (c) Full-length blot image for figure 2b. (d) Full-length blot image for figure 3d. (e) Full-length blot image for figure 3c. (f) Full-length blot image for figure 3e. (g) Full-length blot image for figure 5a. (h) Full-length blot image for figure 5b. (i) Full-length blot image for figure 6a. (j) Full-length blot image for figure 6b. (k) Full-length blot image for figure 6c.(l) Full-length blot image for figure 7a. (m) Full-length blot image for figure 7b. (n) Full-length blot image for figure 7c. (o) Full-length blot image for figure 7d. (p) Full-length blot image for figure 8a. (q) Full-length blot image for figure 8b. (r) Full-length blot image for supplementary figure 1.
